# Supplementary material for: Simple Acoustic Features Can Explain Phoneme-Based Predictions of Cortical Responses to Speech
Source: Curr Biol. 2019 Jun 17;29(12):1924–1937.e9. doi: 10.1016/j.cub.2019.04.067 (PMC6584359; doi:10.1016/j.cub.2019.04.067)
Supplement: Document S1. Figures S1–S7 and Table S1 [file mmc1.pdf]

Current Biology, Volume *29*

Supplemental Information

Simple Acoustic Features Can Explain Phoneme-Based Predictions of Cortical Responses to Speech

Christoph Daube, Robin A.A. Ince, and Joachim Gross

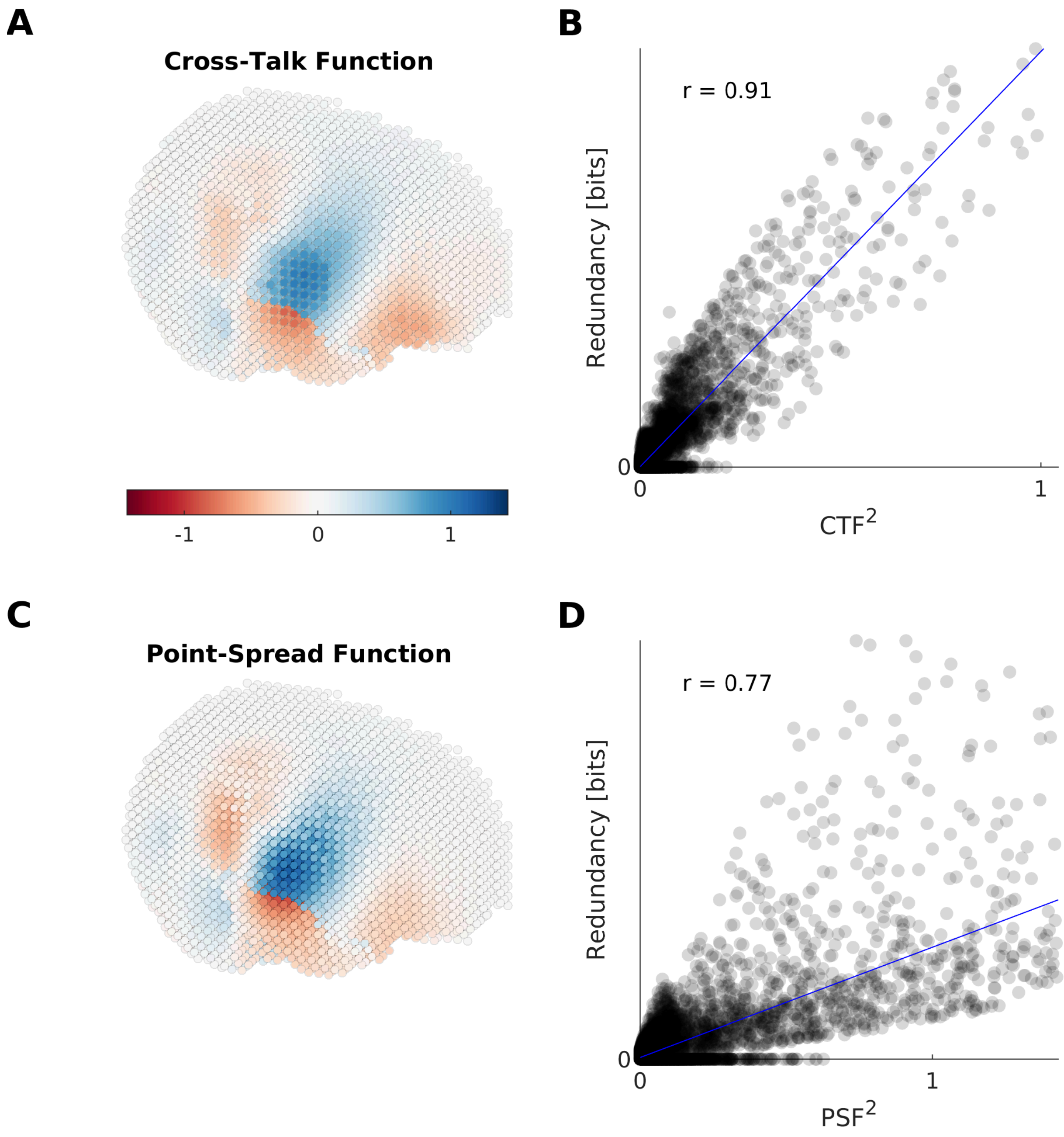

Figure S1: Redundancy is related to Cross-Talk and Point-Spread Functions (related to Figure 2). **(A)** Cross-Talk Function in one exemplary participant (same as in figure 2B). It shows how activity at different grid points leaks into estimates of activity queried at grid point of interest, here at a right AC dipole. **(B)** Relationship of squared Cross-Talk Function (CTF) and Redundancy in exemplary participant. Correlation in top left reports Pearson correlation. The mean of this correlation (Fisher-Z transformed before averaging and retransformed after averaging) was 0.75 (right AC, range: $[0.26, 0.91]$) and 0.68 (left AC, range: $[-0.09, 0.94]$). **(C)** Point-Spread Function in one exemplary participant. It shows how activity at grid point of interest, here at a right AC dipole, leaks into estimates of activity queried at other positions in source space. **(D)** Relationship of squared Point-Spread Function (CTF) and Redundancy in exemplary participant. Correlation in top left reports Pearson correlation. The mean of this correlation (Fisher-Z transformed before averaging and retransformed after averaging) was 0.60 (right AC, range: $[0.16, 0.83]$) and 0.51 (left AC, range: $[-.10, .87]$).

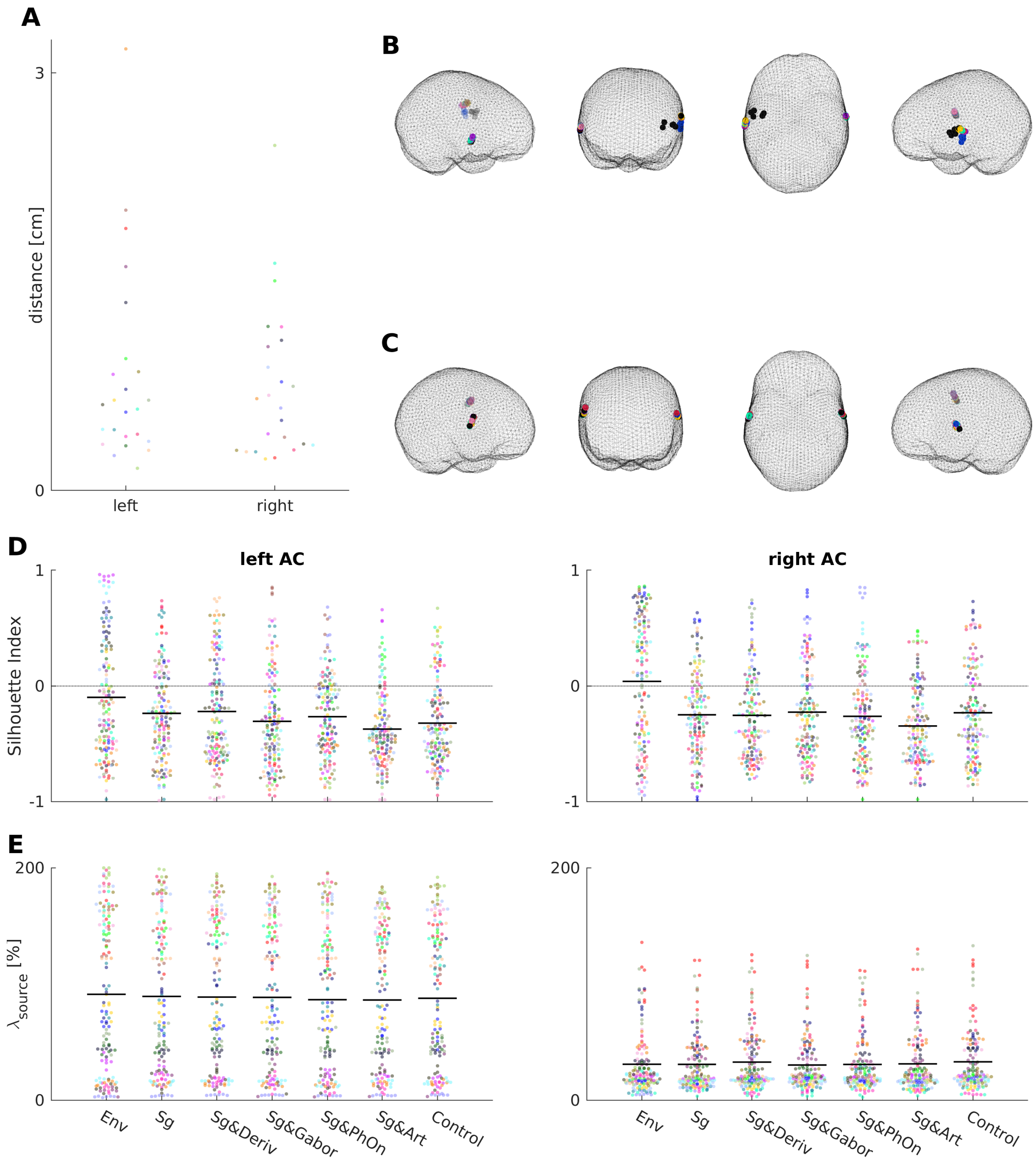

Figure S2: Hyperparameter choices for source model optimisation (related to Figure 3).
(A) Maximum distance between source positions used for test set prediction across all test sets and feature spaces. Each dot is one participant in the respective hemisphere, colour codes participants. **(B)** and **(C)** Positions of all test sets and feature spaces in meshes of individual brain volumes of two exemplary participants. **(B)** shows the participant with the largest maximum distance between chosen source locations and **(C)** the participant closest to the median of maximum distances between chosen source locations. Each dot is one test set, colour codes feature spaces. **(D)** Evaluation of spatial clustering of choices of source positions for different feature spaces using the Silhouette Index. Values close to 1 reflect that the optimisation procedure finds positions in source space that are highly simliar within feature spaces but dissimilar across feature spaces, lower values reflect that the found positions are randomly arranged in source space. Each dot is one test set of one participant, colour codes participants. Black lines denote pooled means. **(E)** Choices of beamformer regularisation hyperparameter λ_{source} for each feature space. Each dot represents one test set of one participant, colour codes participants. Black lines denote pooled means.

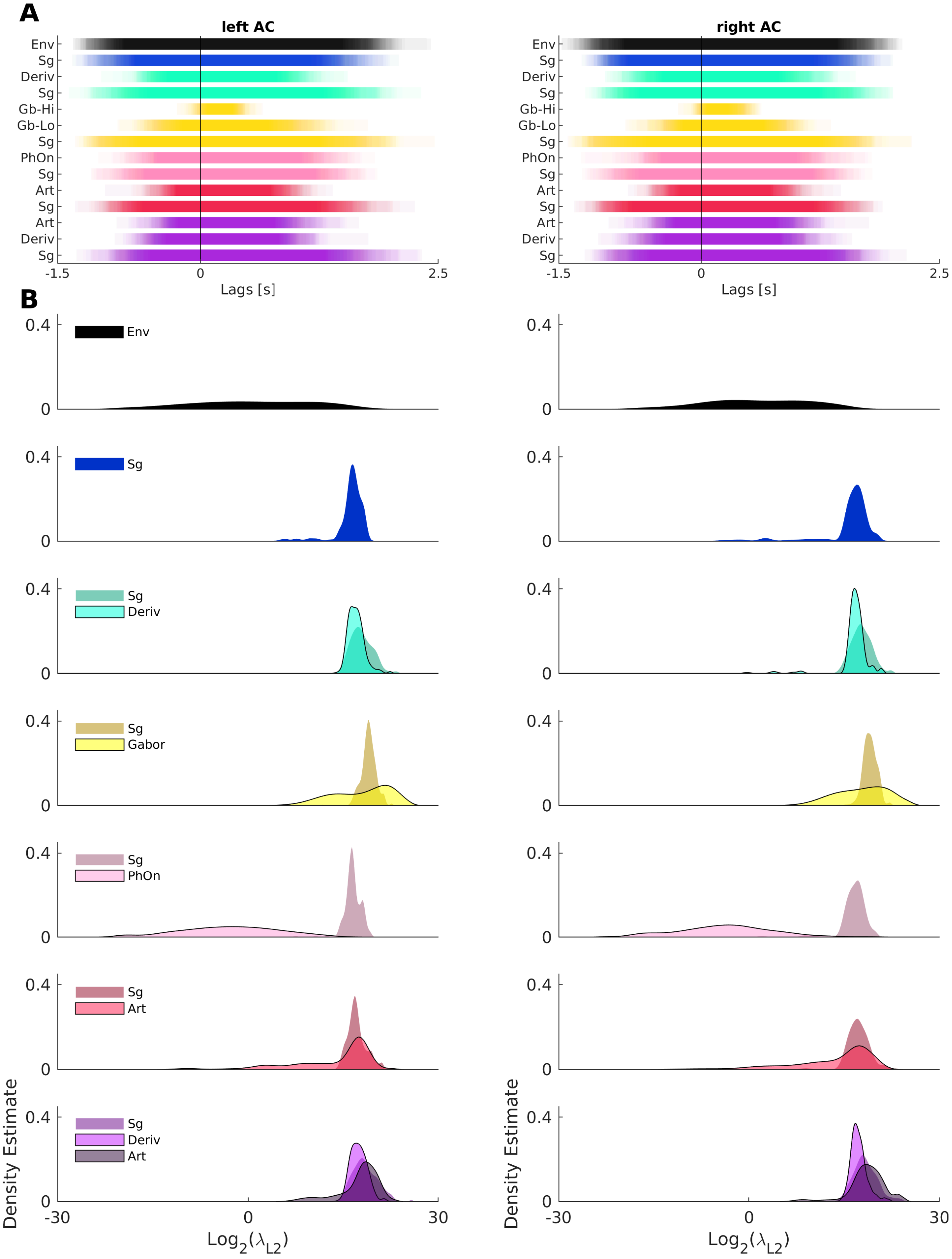

Figure S3: Hyperparameter choices of forward model optimisation differ systematically across feature (sub-)spaces (related to Figure 3).
(A) Choices of temporal extent hyperparameters for each feature (sub-)space. Shown are averages across inner folds used for test set predictions, pooled across participants. Values that were used in all cross-validated models of all participants are plotted as transparent bars ranging from $t_{Min} - t_{Max}$ such that the opacity codes for the number of participants and folds for which the temporal extent was chosen correspondingly. **(B)** Choices of L2 regularisation hyperparameters for each feature (sub-)space. Shown are distributions of choices averaged across inner folds used for test set predictions, pooled across participants. Colours code feature spaces.

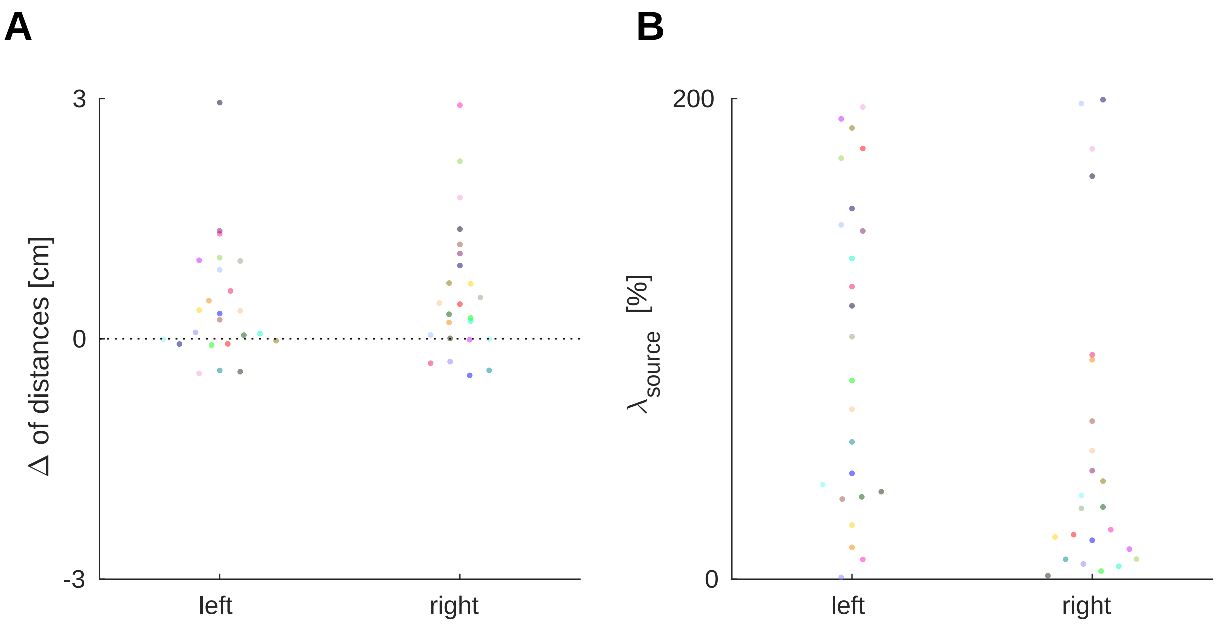

Figure S4: Hyperparameter choices for phoneme related field (PRF) analysis (related to Figure 5).
(A) Maximal euclidean distances of source positions when optimised with regard to model performances across all test sets and feature spaces subtracted from maximal euclidean distances of source positions when positions found when optimising with regard to PRF MI are included. **(B)** Results of optimising sensor covariance regularisation parameter with regard to PRF MI. Colour in **(A)** and **(B)** codes participants.

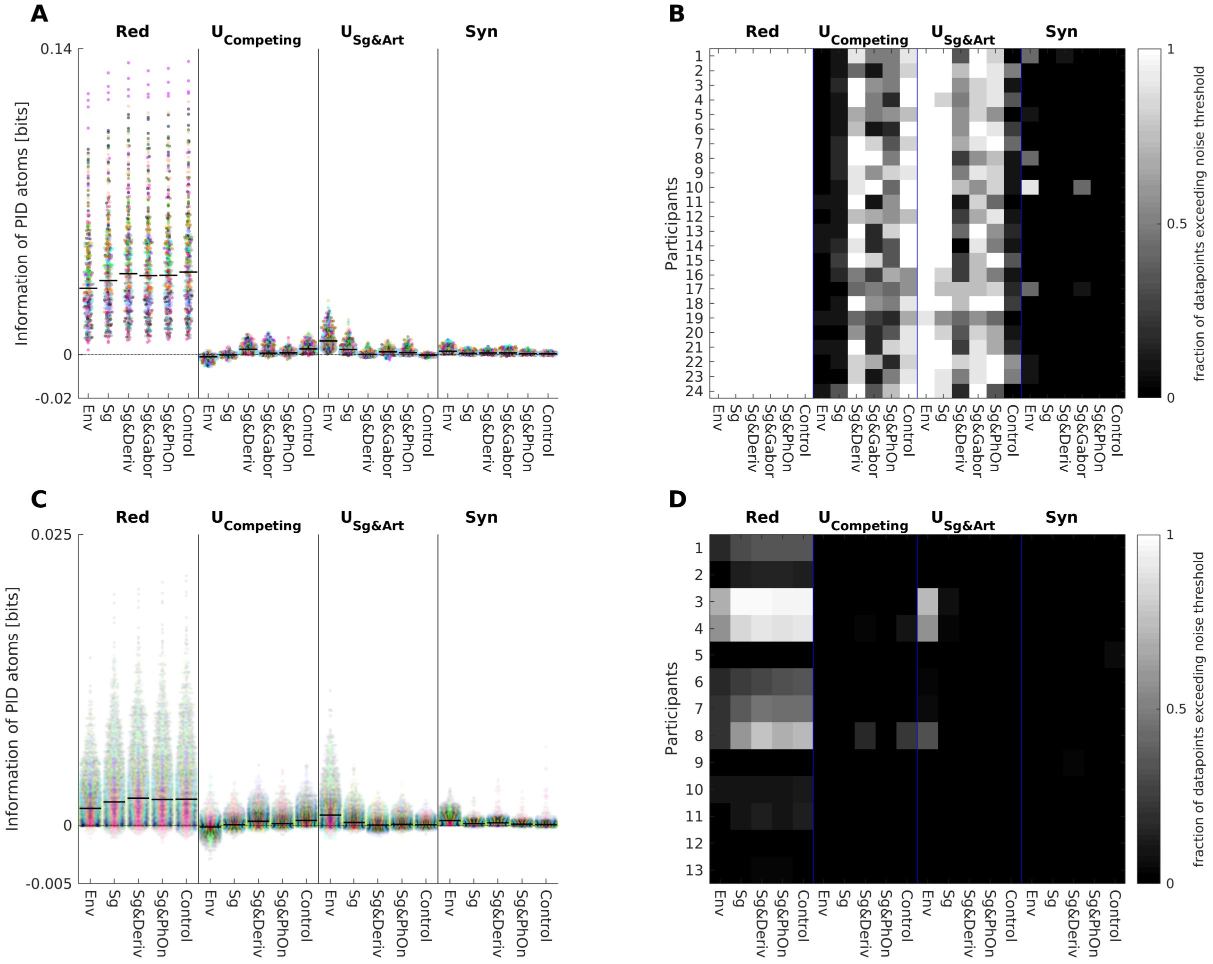

Figure S5: Raw values and comparison to noise thresholds of PID in EEG and MEG (related to Figures 4 and 6).

(A) Raw (unnormalised) PID values (Red: redundancy, U: unique information [of competing feature spaces and of benchmark articulatory feature space] and Syn: synergy) in MEG data from left and right AC. Each colour codes for a single participant, each dot is one test set. Pooled medians are indicated with black lines. **(B)** Comparison of PID values in MEG data to noise thresholds. Image plot shows the fraction of data points (sources, test sets) that exceeded the corresponding noise threshold in each participant and for each feature space and each PID atom. **(C)** Raw (unnormalised) PID values (redundancy, unique information of competing feature spaces, unique information of benchmark articulatory feature space and synergy) in EEG data from all 12 electrodes. Each colour codes for a single participant, each dot is one test set. Pooled medians are indicated with black lines. **(D)** Comparison of PID values in EEG data to noise thresholds. Image plot shows the fraction of data points (sources, test sets) that exceeded the corresponding noise threshold in each participant and for each feature space and each PID atom.

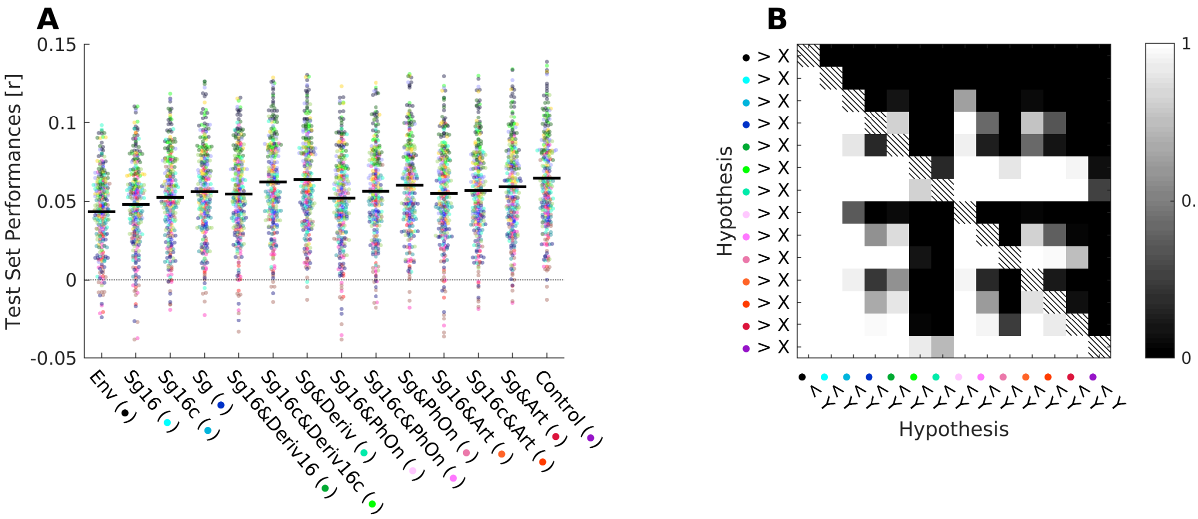

Figure S6: Comparison of 16 channel spectrogram, 16 channel spectrogram with compressive nonlinearity and log-mel spectrogram in EEG data (related to Figure 6).
(A) Raw test set performances of feature spaces. Each dot is one test set of one participant, averaged across electrodes. Colour codes participants. Pooled medians overlaid. Colours in x-axis labelling refer to feature spaces. **(B)** Percent of samples in favour of hypotheses of differences of beta estimates between all feature spaces. Hypotheses are colour coded using the same colour mapping as in x-axis labelling of **(A)**.

The performances obtained using Sg were in general higher than those obtained using $Sg16$, a 16-channel spectrogram modelled after the original study [1]. The combination of $Sg16\&Art$ failed to outperform Sg on its own ($f_{h_1} = 0.2318$).

The overall pattern of performances was largely similar regardless of using Sg or $Sg16$ as the spectrogram. In contrast to the results produced using Sg however, we found that the $Sg16\&Art$ combination outperformed the $Sg16\&PhOn$ combination ($f_{h_1} = 0.9675$).

To assess whether these differences were driven by the compressive linearity included in Sg, we additionally tested a version of $Sg16$ in which we raised its values to the power of 0.3 ("$Sg16c$"). Such nonlinearities are classically included in models of auditory processing, as early as the cochlea [2; 3; 4]. This tweak indeed resulted in a pattern of performances that was largely similar to that obtained with Sg: The combination of $Sg\&Deriv$ did not clearly outperform the combination of $Sg16c\&Deriv16c$ ($f_{h_1} = 0.8191$), and combining $Sg16c\&Art$ was not better than combining $Sg16c\&PhOn$ ($f_{h_1} = 0.6097$).

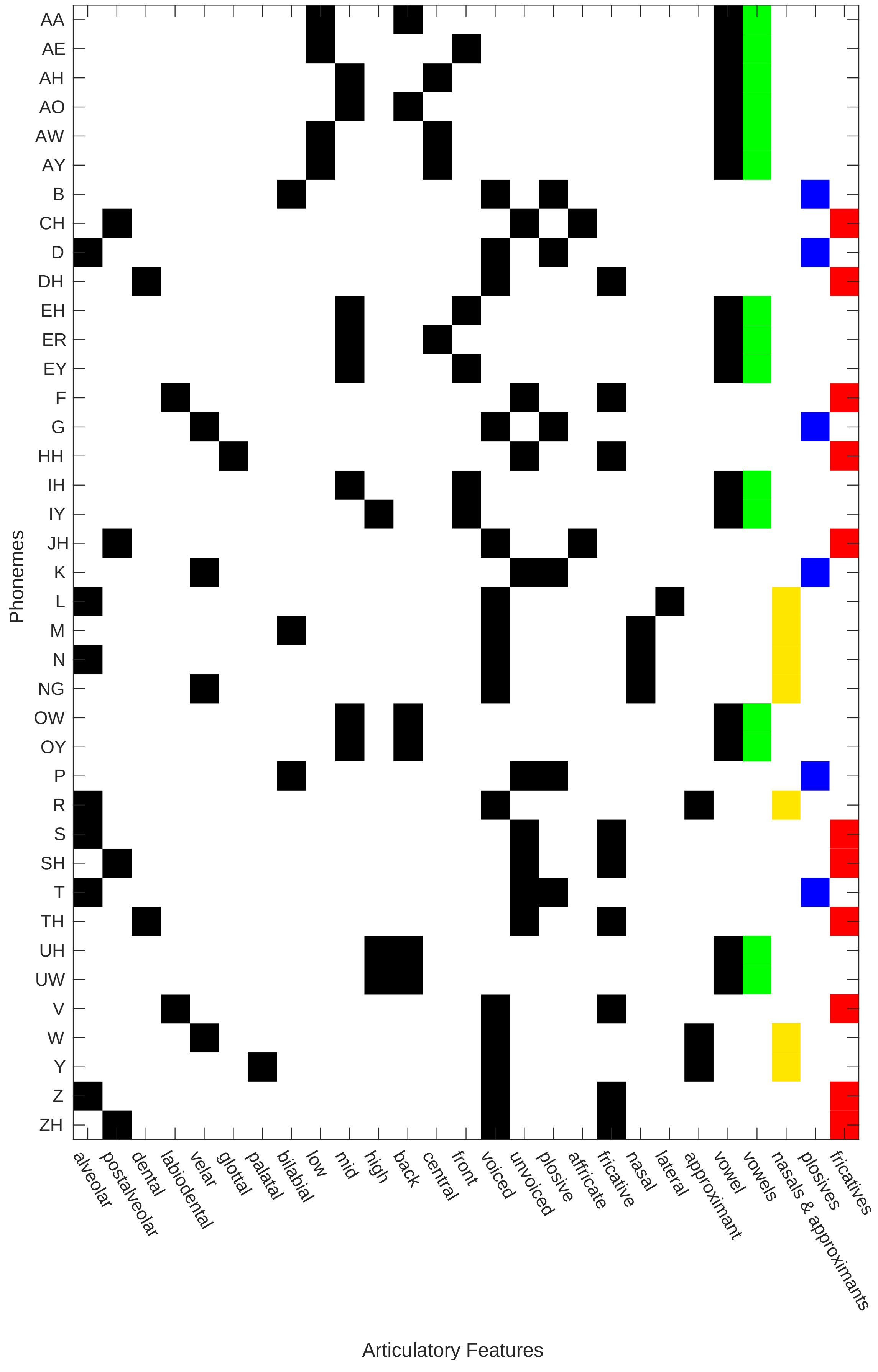

Figure S7: Mapping of phonemes to articulatory features and manners of articulation (related to STAR Methods).
Black and white part shows articulatory features used for forward modelling [1], coloured part shows manners of articulation used for decoding [5].

	initial value	lower boundary	upper boundary
$t_{Min}[s]$	-0.2	-1.5	.5
$t_{Max}[s]$	0.8	0.2	2.5
$\log_{2(\lambda_{L2})}$	19	-30	30
$\lambda_s[\%]$	30	0	200
X_{source}	$X_{max(R^2)\|hemisphere}$	$min(X_{volume\|hemisphere})$	$max(X_{volume\|hemisphere})$
Y_{source}	$Y_{max(R^2)\|hemisphere}$	$min(Y_{volume\|hemisphere})$	$max(Y_{volume\|hemisphere})$
Z_{source}	$Z_{max(R^2)\|hemisphere}$	$min(Z_{volume\|hemisphere})$	$max(Z_{volume\|hemisphere})$

Table S1: Initial values and boundaries for hyperparameters in BADS optimisation (related to STAR Methods).

Supplemental References

S1 Di Liberto, G.M., O'Sullivan, J.A., and Lalor, E.C. (2015). Low-Frequency Cortical Entrainment to Speech Reflects Phoneme-Level Processing. Current Biology *25*, 2457–2465.

S2 Chi, T. and Powen, R. (2005). Multiresolution spectrotemporal analysis of complex sounds. The Journal of the Acoustical Society of America *118*, 887–906.

S3 Verhulst, S., Altoè, A., and Vasilikov, V. (2018). Computational modeling of the human auditory periphery: Auditory nerve responses, evoked potentials and hearing loss. Hearing Research *360*, 55–75.

S4 Biesmans, W., Das, N., Francart, T., and Bertrand, A. (2017). Auditory-Inspred Speech Envelope Extraction Methods for Improved EEG-Based Auditory Attention Detection in a Cocktail Party Scenario. IEEE Transactions on Neural Systems and Rehabilitation Engineering *25*, 402–412.

S5 Khalighinejad, B., da Silva, G.C., and Mesgarani, N. (2017). Dynamic Encoding of Acoustic Features in Neural Responses to Continuous Speech. The Journal of Neuroscience *37*, 2176–2185.
