# Supplementary material for: Simple Acoustic Features Can Explain Phoneme-Based Predictions of Cortical Responses to Speech
Source: Curr Biol. 2019 Jun 17;29(12):1924–1937.e9. doi: 10.1016/j.cub.2019.04.067 (PMC6584359; doi:10.1016/j.cub.2019.04.067)
Supplement: Document S2. Article plus Supplemental Information [file mmc3.pdf]

Article

Current Biology

Simple Acoustic Features Can Explain Phoneme-Based Predictions of Cortical Responses to Speech

Highlights

- 24 participants listened to a 1 h story in MEG
- Encoding and decoding analyses are evaluated and merged using information theory
- Simple acoustic predictors of MEG responses can explain apparent high-level phenomena
- Results are repeated in openly available EEG dataset

Authors

Christoph Daube, Robin A.A. Ince, Joachim Gross

Correspondence

christoph.daube@gmail.com

In Brief

Daube et al. use a data-driven information theoretic analysis of auditory cortex MEG responses to speech to demonstrate that complex models of such responses relying on annotated linguistic features can be explained more parsimoniously with simple models relying on the acoustics only.

Daube et al., 2019, Current Biology *29*, 1924–1937
June 17, 2019 © 2019 The Author(s). Published by Elsevier Ltd.
https://doi.org/10.1016/j.cub.2019.04.067

CellPress

Current Biology
Article

Simple Acoustic Features Can Explain Phoneme-Based Predictions of Cortical Responses to Speech

Christoph Daube,[1,3,*] Robin A.A. Ince,[1] and Joachim Gross[1,2]
[1]Institute of Neuroscience and Psychology, University of Glasgow, 62 Hillhead Street, Glasgow G12 8QB, UK
[2]Institute for Biomagnetism and Biosignalanalysis, University of Münster, Malmedyweg 15, 48149 Münster, Germany
[3]Lead Contact
*Correspondence: christoph.daube@gmail.com
https://doi.org/10.1016/j.cub.2019.04.067

SUMMARY

When we listen to speech, we have to make sense of a waveform of sound pressure. Hierarchical models of speech perception assume that, to extract semantic meaning, the signal is transformed into unknown, intermediate neuronal representations. Traditionally, studies of such intermediate representations are guided by linguistically defined concepts, such as phonemes. Here, we argue that in order to arrive at an unbiased understanding of the neuronal responses to speech, we should focus instead on representations obtained directly from the stimulus. We illustrate our view with a data-driven, information theoretic analysis of a dataset of 24 young, healthy humans who listened to a 1 h narrative while their magnetoencephalogram (MEG) was recorded. We find that two recent results, the improved performance of an encoding model in which annotated linguistic and acoustic features were combined and the decoding of phoneme subgroups from phoneme-locked responses, can be explained by an encoding model that is based entirely on acoustic features. These acoustic features capitalize on acoustic edges and outperform Gabor-filtered spectrograms, which can explicitly describe the spectrotemporal characteristics of individual phonemes. By replicating our results in publicly available electroencephalography (EEG) data, we conclude that models of brain responses based on linguistic features can serve as excellent benchmarks. However, we believe that in order to further our understanding of human cortical responses to speech, we should also explore low-level and parsimonious explanations for apparent high-level phenomena.

INTRODUCTION

Speech perception is often conceptualized as a hierarchical process [1, 2]. The human brain is assumed to extract semantic meaning from a highly dynamic sound pressure signal via a cascade of transformations that create increasingly abstract representations of speech. It is well established that perceived speech sounds are first decomposed into a spectrally resolved representation at the cochlea. Various structures along the subcortical auditory pathway are believed to then undertake further processing steps [3, 4]. However, considerable uncertainty remains about exactly how sound is represented in the auditory cortex (AC) [5].

One way to gain further insight into human speech processing is to employ encoding models. These models aim to predict the time series of recorded neural data from the waveform of the presented stimulus. A popular framework for encoding models organizes this in two steps [6, 7]. In the first step, the stimulus material undergoes nonlinear transformations into various sets or spaces of features. These features capture hypotheses about the cortical computations that are performed on the input signal. In the second step, a linear mapping of these feature spaces onto the neuronal responses is obtained to evaluate the utilized hypotheses in terms of out-of-sample prediction performance. In this way, data-rich, naturalistic listening conditions of a relatively long duration can be exploited, considerably improving a model's validity over isolated and artificial experimental paradigms [8, 9]. Recent results demonstrate the applicability of this approach across various neuroimaging modalities and research questions [10–20].

A compelling finding obtained with this approach is that predictions of cortical responses as measured by electroencephalography (EEG) [10] or functional magnetic resonance imaging (fMRI) [12] using acoustic feature spaces can be improved by additionally considering so-called articulatory feature spaces. The latter originate from the linguistic concept of representing a language with a set of minimal contrastive units, called phonemes. However, superior temporal regions are known to selectively respond to subgroups of phonemes rather than to individual phonemes [21]. Therefore, the full phoneme set is usually reduced by mapping each phoneme to its corresponding vocal gestures ("articulatory features"), such as the voicing, tongue position, or place and manner of articulation. Recently, it was shown that these manners of articulation can also be decoded from EEG data time-locked to phoneme onsets in continuous speech stimuli [22]. Encoding and decoding analyses based on articulatory feature spaces are thus interpreted as concordantly capturing a faculty called "pre-lexical abstraction" [23], i.e., a transformation of continuous physical properties of

 © 2019 The Author(s). Published by Elsevier Ltd.
This is an open access article under the CC BY license (http://creativecommons.org/licenses/by/4.0/).

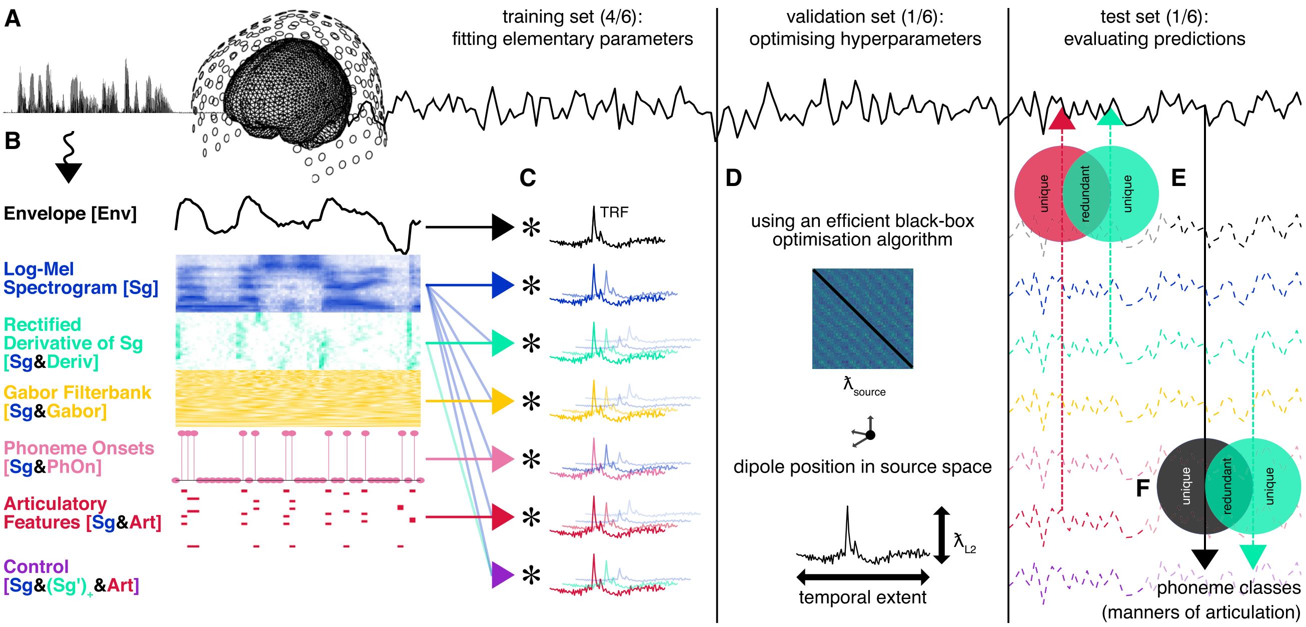

Figure 1. Study Concept and Design

(A) Magnetoencephalography (MEG) data were recorded while participants (n = 24) listened to a story of 1 h duration.

(B) The speech waveform was then nonlinearly transformed into various feature spaces.

(C) These feature spaces were used to predict neuronal responses using (multivariate) temporal response functions ((m)TRFs) in a nested cross-validation framework. The majority of the data were used to fit the (m)TRFs.

(D) Hyper-parameters controlling the (m)TRFs (separately for each feature (sub-)space, hemisphere, and participant: temporal extent and L2 regularization) and the MEG source reconstruction (sensor covariance matrix regularization and position of dipoles in source space) were optimized on separate validation data.

(E) The predicted responses of the encoding model (dashed lines) were evaluated on unseen test data by asking to which degree a benchmark feature space that relied on articulatory features was redundant with competing, acoustic feature spaces using partial information decomposition (PID).

(F) Additionally, four classes of phonemes were decoded from phoneme-locked observed and predicted MEG responses. PID was used to determine to which degree the predictions of the encoding models contained the same information about phoneme classes as the observed data.

the waveform to speech-specific, categorical, and invariant units of perception.

However, the transformation of speech stimuli into articulatory features comes with certain critical caveats. Most importantly, this representational format of speech is based on concepts that humans have agreed on to talk about language. And while a match of such linguistic constructs with physiological responses is conceivable, it is a potentially biased and specific hypothesis with a range of alternatives [1, 24–26].

Moreover, the partly arbitrary mapping of phonemes to articulatory features provides a low degree of computational specification. As such, models that use articulatory features could be considered to be so-called "oracle models," which rely on information that is not available to the individual's brain being modeled [27].

Additionally, current implementations of this transformation rely on a semi-automated, forced alignment of a textual transcription to the sound wave of the stimulus material. While such alignment methods incorporate a high degree of computational sophistication, the task they solve is not a good model of the task that the listening brain faces. This compromises the usefulness of the intermediate representations generated by such alignments to serve as candidate features to predict brain responses, such that usually only the final output is used. It thus remains unclear whether the level of complexity implied by the final articulatory features is actually necessary.

These caveats thus raise an important question. Can the gain in prediction performance that is reportedly provided by articulatory features be explained by alternative features that are based on computationally more specified, physiologically plausible, and possibly less complex transformations of stimulus acoustics? The extent to which this were the case would indicate how much of the predictive gain that is provided by articulatory features is attributable to the generic, feedforward processing of an acoustic stimulus that is not specific to speech processing.

When choosing such acoustic feature spaces, one can proceed in different directions. One possibility is that, in order to explain the same variance as models based on articulatory features, the characteristic spectrotemporal patterns that define the phoneme subgroups are needed. Correspondingly, one could extract such abstract information from the spectrogram with suitable filters. A physiologically inspired candidate feature space is the Gabor-filtered spectrogram, which interestingly improves the performance of automatic speech recognition (ASR) software when used as input features [28]. With this generic class of spectrotemporal kernels, one can describe several acoustic patterns that dissociate groups of phonemes. Examples include the spectral distance between formants, as captured by filters of different spectral modulation, and formant transitions, as captured by filters of joint spectrotemporal

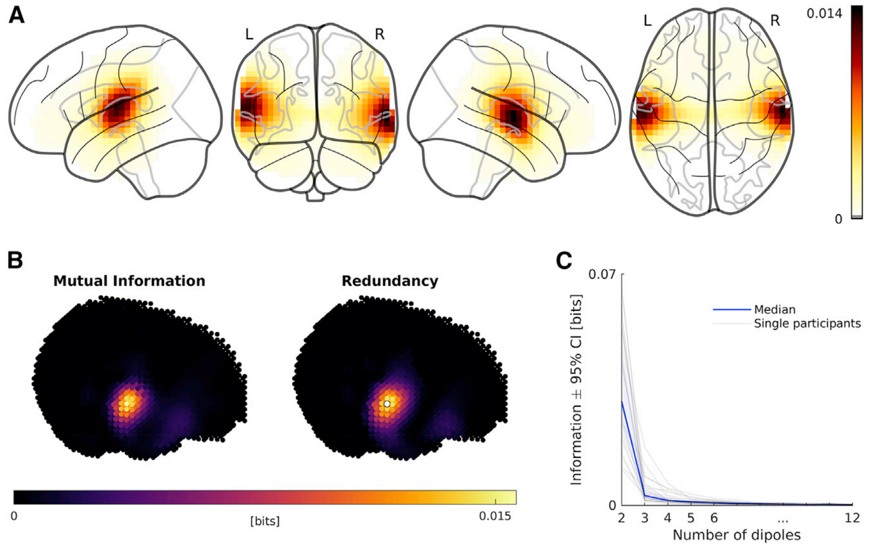

Figure 2. Identification and Characterization of Story-Responsive Regions in Source Space

(A) Grand average story responsivity (variance of source-reconstructed brain activity recorded during first presentation explained by activity recorded during second presentation of the last block). Each image shows different viewing angles on the same data.

(B) (Left) Story responsivity using mutual information (MI). Plot shows MI of activity in the first repetition of the last block about activity in the second repetition of the last block. (Right) Shared information (redundancy) of activity at bilateral story-responsivity peaks in the first repetition and activity in the first repetition at each other grid point about activity at these other grid points in the second repetition. See Video S1 for further explanation. Data from one exemplary participant are shown.

(C) Unique information added by sources additional to the bilateral story-responsivity peaks.

See also Figure S1.

modulation. Although this feature space is long established in encoding and decoding models of the human and animal midbrain and ACs [7, 13, 29–34], it has yet to be applied to magneto- and electroencephalography (MEEG) data.

Another possibility is that the performance boost provided by articulatory features is instead attributable to their correlation with simpler acoustic properties. It has repeatedly been observed that neuronal responses from bilateral superior temporal regions are particularly sensitive to acoustic edges [35–40]. Features that extract these onsets from envelope representations via a half-wave rectification of the temporal gradient of time-varying energy have been used in several studies [36, 41, 42]. Features that rely on the temporal gradient also capture the relationship of neighboring time points, which contain information present in MEEG data across a range of different analyses [43]. It is thus interesting to assess the degree to which the gain in prediction performance that is provided by articulatory features can be explained by such onset features.

In this study, we examined these two possible explanations by comparing the predictive power of different acoustic feature spaces to that of an annotated articulatory feature space. We performed these investigations on an magnetoencephalogram (MEG) story-listening dataset of 1 h duration per participant in a rigorous, data-driven approach (see Figure 1). A nested cross-validation framework [44] was used to delegate the choice of model settings to a recent optimization algorithm [45]. We thus allowed encoding models based on different feature spaces the same chances to find optimal parameter combinations with a minimum of *a priori* information, while minimizing the risk of overfitting. We then applied partial information decomposition [PID, 46] to assess the degree to which the predictions of acoustic feature-based models shared information about observed recordings with those of articulatory feature-based models and to assess the degree to which these feature spaces contained unique predictive information. This flexible theoretic framework also allowed us to quantify to what extent the information about manners of articulation decodable from phoneme-evoked responses could be accounted for by the predictions of our encoding models. Lastly, since MEG and EEG data can reflect different neuronal processes [47, 48], we performed similar analyses on a publicly available EEG story-listening dataset [49]. Using this approach, we found that apparent encoding and decoding signatures of high-level pre-lexical abstraction could be explained with simple low-level acoustic models.

RESULTS

Speech Tracking in Bilateral ACs

First, we characterized where in MEG source space we could find robust responses related to speech processing and also the spatial resolution that these responses could be studied at. To identify regions in source space where MEG responses were repeatably activated by the stimulus ("story-responsive" regions [12, 50]), we correlated source-reconstructed, full-brain responses to one chapter of the story with the responses to its repeated presentation. These correlations peaked in regions that agree with the typical localization of the bilateral ACs (Figure 2A).

Instead of falling off sharply, the story responsivity decreased gradually with increasing distance from these peaks. However, we expected that querying activity from different locations within these story-responsive regions would yield highly similar (i.e., redundant) time series since the spatial resolution of MEG inverse solutions is inherently limited [51]. To avoid an unnecessary computational burden for the later modeling, we therefore explored how much of the repeatable activity we could explain with dipoles at the two bilateral story responsivity peaks and also how much we could explain by considering further dipoles at different locations. To do so, we implemented an iterative information theoretic approach based on PID (see Video S1 and STAR Methods for a detailed description). This approach revealed that indeed one source per hemisphere could account for most of the spatial spread of the story responsivity. The individual maps of story responsivity correlated highly with maps of redundancy (average Pearson correlation across participants:

Table 1. Feature Spaces

Shorthand	Name	Dimensionality	Description
Env	envelope	1	sum across channels of Sg [36, 37, 52]
Sg	log-mel spectrogram	31	spectral decomposition of time-varying stimulus energy in 31 mel-spaced bands with logarithmic compressive nonlinearity [3, 28, 53]
Sg & Deriv	log-mel spectrogram and half-wave rectified temporal derivatives of individual spectrogram channels	31 + 31	Sg and positive temporal rate of change of power in each channel of Sg [18, 36, 40, 42, 43]
Sg & Gabor	log-mel spectrogram and Gabor-filtered spectrogram	31 + 455	Sg and decomposition of Sg according to spectral, temporal, and joint spectrotemporal modulations [28–31, 33, 34, 53]
Sg & PhOn	log-mel spectrogram and annotated phoneme onsets	31 + 1	Sg and unit impulses at the beginning of each annotated phoneme [18]
Sg & Art	log-mel spectrogram and articulatory features of each phoneme, "benchmark feature space"	31 + 23	Sg and 23 channels with unit impulses at the beginning of each phoneme characterized by the corresponding vocal gesture [10, 12]
Control	log-mel spectrogram, half-wave rectified temporal derivatives of individual spectrogram channels and articulatory features of each phoneme	31 + 31 + 23	combination of Sg, Deriv, and Art feature spaces

0.89; range: 0.80–0.97; Figure 2B). As such, the information that activity at additional grid points carried about the activity recorded during the second presentation of the same chapter largely overlapped with the information that could be obtained from activity at the bilateral peaks. Correspondingly, the amount of information contributed by sources additional to the bilateral peaks fell off in a characteristic L-shaped curve (Figure 2C). This was largely attributable to measures of leakage of the spatial filters, such as their cross-talk and point-spread functions (see Figure S1 for details).

Based on these results, we subsequently analyzed one source location per hemisphere, since this single location could capture the repeatable signal that stems from the bilateral ACs. Note, however, that in the following modeling, the exact location of these two sources was not fixed but instead was optimized independently for each tested feature space.

Predictive Power of Feature Spaces

The main goal of this study was to compare the cross-validated performance of linear models that were trained to predict relevant parts of the MEG responses from different sets or "spaces" of features extracted from the speech stimulus. We assessed this using the Pearson correlation between observed and predicted MEG time courses. The central question we investigated was to what degree can purely acoustic feature spaces achieve the performance of a benchmark feature space (namely, spectrograms and annotated articulatory features combined [10])? Crucially, our modeling approach ensured that the settings of our models ("hyper-parameters") could flexibly adapt to each different feature (sub-)space, individual participant, and to each hemisphere (see STAR Methods for a detailed description). The hyper-parameters operated on the predictors, the model, and also the MEG responses such that, for example, the exact position of the dipole in source space was optimized for each feature space (see Table 1 for an overview over all feature spaces used in this study). This gave each feature space the same chance to optimally predict the MEG responses within our bilateral sources linear modeling framework.

The performances of our models exhibited relatively large inter-participant variability and comparatively low variability across feature spaces (Figure 3A). To focus on the systematic differences across the feature spaces, we used Bayesian hierarchical linear modeling [54] and separated the overall effects of different feature spaces from effects attributable to participants, hemispheres, and cross-validation folds. We extracted the samples of the posterior distributions of the regression coefficients ("βs") of interest. We then subtracted the samples that referred to the benchmark feature space from those referring to the other competing feature spaces. From the resulting posteriors of differences (Figure 3B), we could determine the fraction of samples above or below zero, i.e., in the direction of the corresponding hypotheses (f_{h_1}). We repeated this for all other possible comparisons between the feature spaces (Figure 3C).

Initially, we were interested in whether we could replicate the previously reported increase in prediction performance when combining linguistically motivated articulatory features with spectrograms (Sg & Art, red vertical line; Figure 3B) over spectrograms alone (Sg, blue) in our data. Indeed, we found a large fraction of samples of the posterior of differences in favor of a successful replication (mean of the difference in Pearson correlation Δ = 0.0093; f_{h_1} = 0.9994). This allowed us to test whether various alternative feature spaces could achieve a similar gain in performance in order to investigate the origin of the improved prediction achieved using articulatory features.

We first investigated spectrotemporal Gabor patterns, which can be used to dissociate several phonemic groups [28], because the articulatory feature space might have benefitted from describing responses that are specific to phoneme subgroups. In combination with the spectrogram, which directly accounted for time varying sound energy, this feature space (Sg & Gabor, yellow) achieved a comparable gain in prediction performance over the spectrograms alone (Δ = 0.0098; f_{h_1} =

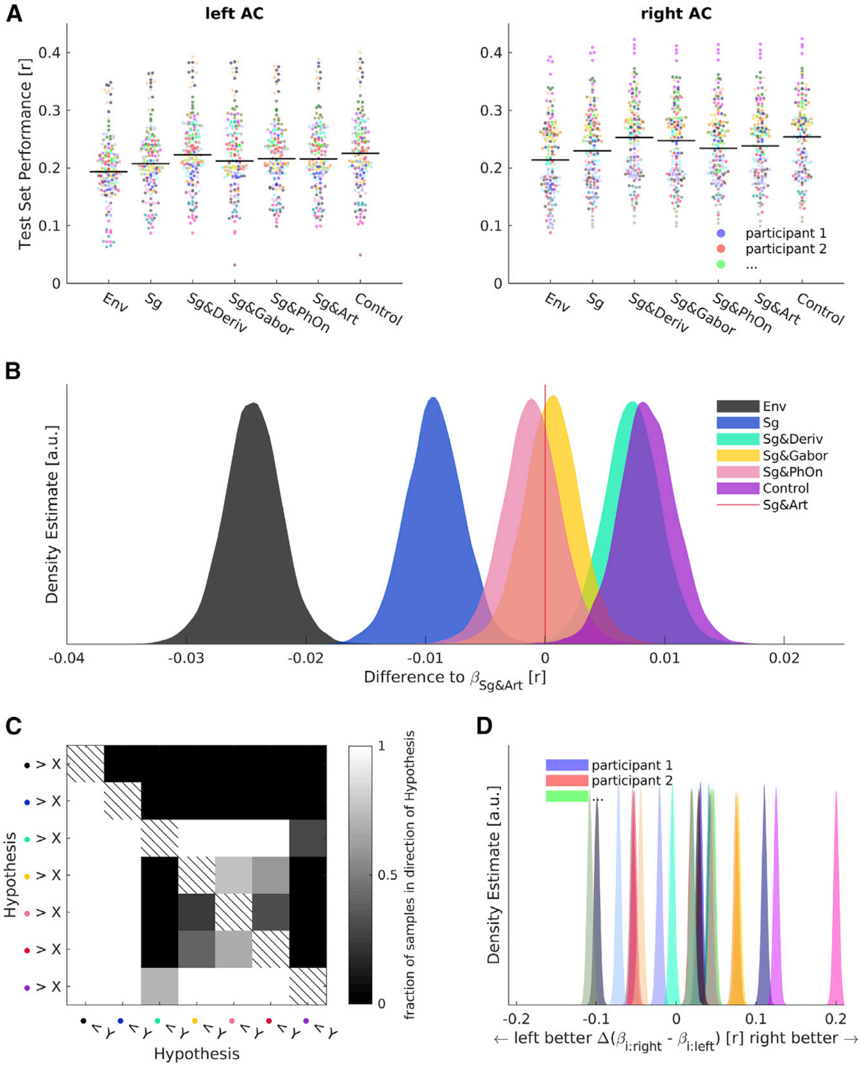

Figure 3. Evaluating the Performance of Different Feature Spaces

(A) Raw test set performances in the left and right auditory cortex (AC) for models based on different feature spaces shown on the horizontal axis. See Table 1 for an explanation of the feature spaces and their shorthand notations. Each color codes for a single participant (n = 24); each dot is one test set. Pooled medians are indicated with black lines.

(B) Samples from the posterior distribution of differences of beta estimates (competing feature spaces minus benchmark Sg & Art feature space, results left of the red line thus reflect that the Sg & Art feature space has a higher performance; results right of the red line indicate that the competing feature space has a higher performance). Feature spaces are color coded as indicated.

(C) Percentage of samples in favor of hypotheses of differences of beta estimates between all feature spaces. Hypotheses are color coded according to the same color mapping as in (B), which corresponds to the bottom row and right column of the matrix shown here.

(D) Samples from posterior distribution of differences of beta estimates of individual participant's right ACs minus left ACs. Color mapping is the same as in (A).

See also Figures S2 and S3.

0.9994). Its performance was on par with the benchmark feature space (Sg & Art), i.e., it was only negligibly better (Δ = 0.0006; f_{h_1} = 0.5960). This feature space thus achieved a similar performance to that of the linguistically motivated feature space but did so without requiring linguistic concepts. Instead, it was physiologically motivated and computationally fully specified.

However, we also wanted to explore simpler models to determine the level of complexity that would be required to optimize prediction. Sound onsets offer a promising candidate for a neurally relevant, low-dimensional auditory feature [36, 39, 40, 43]. As a first test of this hypothesis, we reduced the articulatory features to phoneme onsets (Sg & PhOn, pink). This model outperformed the spectrograms in a similar way to Sg & Art (Δ = 0.0081; f_{h_1} = 0.9983), indicating that the performance increase obtained with articulatory features originates from the timings of the phoneme onsets, and not the identity of different phoneme subgroups.

The phoneme onsets were, however, still an abstracted representation of the stimulus resulting from transcription alignment, with an unclear relation to the original acoustics. One way to derive a signal representing sound onsets directly from speech acoustics is by half-wave rectification of the first derivative of the time-varying stimulus energy [36]. This quantifies positive rates of change, i.e., increases in the stimulus amplitude. We had found that spectrally resolving the amplitude using spectrograms (Sg, blue) outperformed the broadband envelope (Env, black; Δ = 0.0152; f_{h_1} = 1). We therefore computed the positive rate of change of energy of the individual channels of the spectrogram. Combined with the spectrogram features, this model (Sg & Deriv, turquoise; Figure 3B) outperformed the benchmark feature space (Δ = 0.0073; f_{h_1} = 0.9972). It also outperformed the combination of spectrograms and Gabor-filtered spectrograms (Δ = 0.0067; f_{h_1} = 0.9958). Thus, a relatively simple acoustic feature space that focused on acoustic edges not only equaled the benchmark but surpassed it.

As a first test whether these best acoustic features could account for the same information as the articulatory features, we also tested a combination of them (Control, purple). The improvement of this combination of three feature subspaces over the best acoustic feature space was negligible (Δ = 0.0013; f_{h_1} = 0.7078). This indicated that the articulatory features are not needed for an optimal prediction of the MEG responses.

We also explored the lateralization of the performances by evaluating within-participant differences across hemispheres independent of feature spaces (Figure 3D). We found that the posterior distributions of hemispheric beta differences were narrow for individual participants but exhibited a broad range of means within our sample. Some participants' responses were easier to

explain in the left AC, others were easier to explain in the right AC, and for some there were no strong lateralization effects.

Taken together, these results demonstrate that the gain in prediction performance obtained by combining articulatory features with spectrograms can be replicated in MEG data. However, a similar or even larger gain can be obtained by using algorithmically specified and generic acoustic features that capitalize on acoustic edges. Their performance in turn could not be improved by combining them with articulatory features. Next, we wanted to reveal in more detail how the precise information about the MEG predicted by the competing feature spaces was related to the information predicted by the benchmark articulatory features: were the similar levels of performance driven by the same or by different predictive information?

Shared and Unique Information of Articulatory and Acoustic Features

Even if two models have the same predictive power, both higher than a reference model, each could offer improved performance based on different information (i.e., by better predicting different periods of the speech signal) or the same information (i.e., by better predicting the same periods of speech). The PID information theoretic framework [46, 55] (see STAR Methods for details) provides a means to dissociate these situations. We used it to address two questions: (1) to which degree is the information carried by the acoustic-feature-based predictions shared (redundant) with that carried by predictions based on the benchmark articulatory features? And (2) to which degree do the predictions from each feature space contain unique information? If the benchmark features could be explained by the acoustic alternatives, then the results would be characterized by (1) a high degree of redundancy and (2) a low amount of unique information left to the benchmark articulatory features. Such a finding would suggest that the two feature spaces predict the same parts of the response in the same way.

To investigate this question, we retrained all models with their source-space-related hyper-parameters fixed to the values that were found to be optimal for the benchmark articulatory features. We then considered separate, pairwise PIDs, where each acoustic feature space was compared to the benchmark articulatory feature space (Figure 4). To make the resulting quantities more easily interpretable, we normalized the resulting redundant and unique information by the marginal mutual information (MI [43] a non-parametric measure of the relationship between variables) of the benchmark features and the observed MEG. We then statistically analyzed these values using Bayesian hierarchical models similar to our analyses of the raw performances, focusing again on the regression coefficients that modeled the effects of feature spaces.

The acoustic features with the best prediction performance, Sg & Deriv, were indeed also highly redundant with the benchmark articulatory features, reaching ~100% of the marginal MI provided by Sg & Art about the observed MEG (mean of the corresponding effect = 0.99; 95% credible interval [CI] = 0.98–1.01). The same was the case when combining the best acoustic features with the articulatory features (Control: mean = 1.01; 95% CI = 0.99–1.02). Furthermore, we observed more unique information present in the acoustic feature space (mean = 0.07; 95% CI = 0.06–0.09) than in the benchmark articulatory feature space (f_{h_1} = 1), in which the unique information was distributed around 0 (mean = 0.01; 95% CI = −0.01 to 0.02). This means that all of the predictive information of the benchmark Sg & Art model was included in the predictions of the Sg & Deriv model. There was no unique information available in the Sg & Art prediction that a Bayesian optimal observer could not have extracted from the Sg & Deriv model.

Last, the information about the MEG responses only available from a joint consideration (i.e., synergy) of the benchmark articulatory features and the best acoustic features had a negligible effect size that was two orders of magnitude lower than that of the redundancy and failed to surpass a permutation-based noise threshold (see Figure S5). These results agreed with the finding that a combination of the best acoustic feature spaces and the articulatory features did not have a better prediction performance than the best acoustic features (see previous section).

A relatively high normalized redundancy close to 100% was also achieved by Sg & PhOn (mean = 0.97; 95% CI = 0.96–0.99). In addition, Sg & PhOn provided a weak amount of unique information (mean = 0.03; 95% CI = 0.01–0.05) and left a very similar amount of unique information to the benchmark articulatory feature space (mean = 0.03; 95% CI = 0.01–0.04). The annotated onsets thus provide most of the information that the benchmark features provide about the observed MEG.

A very similar pattern was found for the second-best acoustic features, Sg & Gabor. These features also achieved a relatively high redundancy (mean = 0.96; 95% CI = 0.95–0.97) but one that was lower than that of the best acoustic features (f_{h_1} = 0.9988). Sg & Gabor also provided a weak amount of unique information (mean = 0.04; 95% CI = 0.02–0.05) and left a very similar amount of unique information to the benchmark articulatory features (mean = 0.04; 95% CI = 0.02–0.06). We conclude that this high-dimensional acoustic feature space included both relevant and many irrelevant dimensions. The increase in the separability of the different spectrotemporal patterns that refer to different phoneme subgroups [28] is thus less important than the sound energy patterns that are contained in the best acoustic feature space.

Finally, as expected from their comparably low prediction performances, the remaining feature spaces (Env and Sg) exhibited redundancies that were lower than that of the previously mentioned feature spaces (both f_{h_1} = 1). They also left considerable amounts of unique information to the benchmark feature space while providing no substantial positive unique information themselves (mean of Env = −0.03; 95% CI = −0.05 to −0.02; mean of Sg = 0.00; 95% CI = −0.02 to 0.02).

On a group level, all of these patterns were highly similar between left and right ACs.

Thus, the best acoustic features achieve their improved prediction performance over spectrograms alone by explaining the same parts of the responses that the benchmark articulatory features explain, and they additionally explain parts that the linguistic features do not, while a joint consideration of both feature spaces does not add meaningful extra information.

Phoneme-Evoked Dynamics of Observed and Predicted Time Series

As recently demonstrated, four manners of articulation of phonemes can be decoded from EEG data [22]. We next assessed

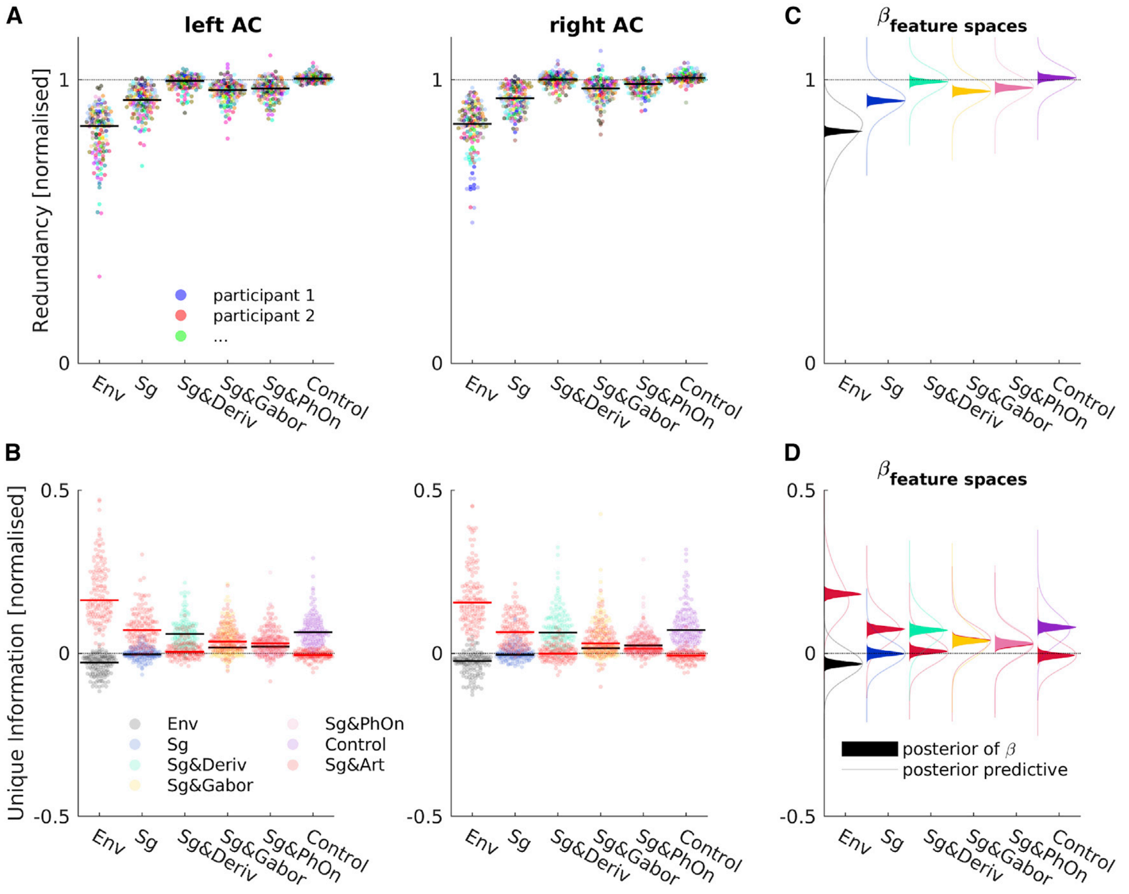

Figure 4. Shared and Unique Contributions of Articulatory and Competing Features

(A) Normalized redundancy in left and right auditory cortices (ACs). Each color codes for a single participant (n = 24). Each dot is one test set of one participant; black and red lines show pooled medians.

(B) Normalized unique information of benchmark articulatory features and competing features in left and right AC. Colors code for a feature space, as shown. Each dot is one test set of one participant; black and red lines show pooled medians.

(C and D) Modeling of redundancy and unique information results, respectively. Filled areas show density estimates of posterior distributions of estimates of betas of feature spaces. Lines show density estimates of samples from posterior predictive distribution of the respective condition. Color coding of feature spaces is the same as in (B).

See also Figure S5.

whether this decoding was possible in our MEG data and the degree to which our encoding models could account for this phenomenon.

For this decoding analysis, we re-optimized the dipole position and sensor covariance matrix regularization parameters of the spatial filters. We did this by using black-box optimization, as before [45], only this time with respect to the MI between MEG data epoched to phoneme onsets and the manner of articulation of each phoneme (four discrete phoneme classes were used: vowels, nasals, plosives, and fricatives; see Figure S7). The MI was calculated separately for each time point in the extracted phoneme epochs. For optimization, we subsequently summed the MI across time points. In most cases, the positions found in this re-optimization were very similar to those found before (Figure S4A).

At the corresponding source locations, we found characteristic responses to the four manners of articulation (i.e., the four phoneme classes used; see Figure 5A). We then retrained our encoding models based on all feature spaces with the source-level parameters fixed to the values found when optimizing for MI between MEG data epoched to phoneme onsets and the manners of articulation. In the cross-validated predictions of these retrained models, we observed phoneme-locked responses that were very similar to those obtained with observed MEG data (Figure 5A, right).

Correspondingly, we observed a sustained pattern of MI following the phoneme onsets in bilateral ACs for the observed data (Figure 5B). We found very similar patterns of MI between manners of articulation and predicted phoneme-related fields, with values roughly an order of magnitude higher for the predictions. On average, this result pattern did not substantially differ between either of the two hemispheres or between the different feature spaces.

Together, our results thus show that the decoding of these manners of articulation was replicable in the observed MEG data and in the MEG data predicted by our models.

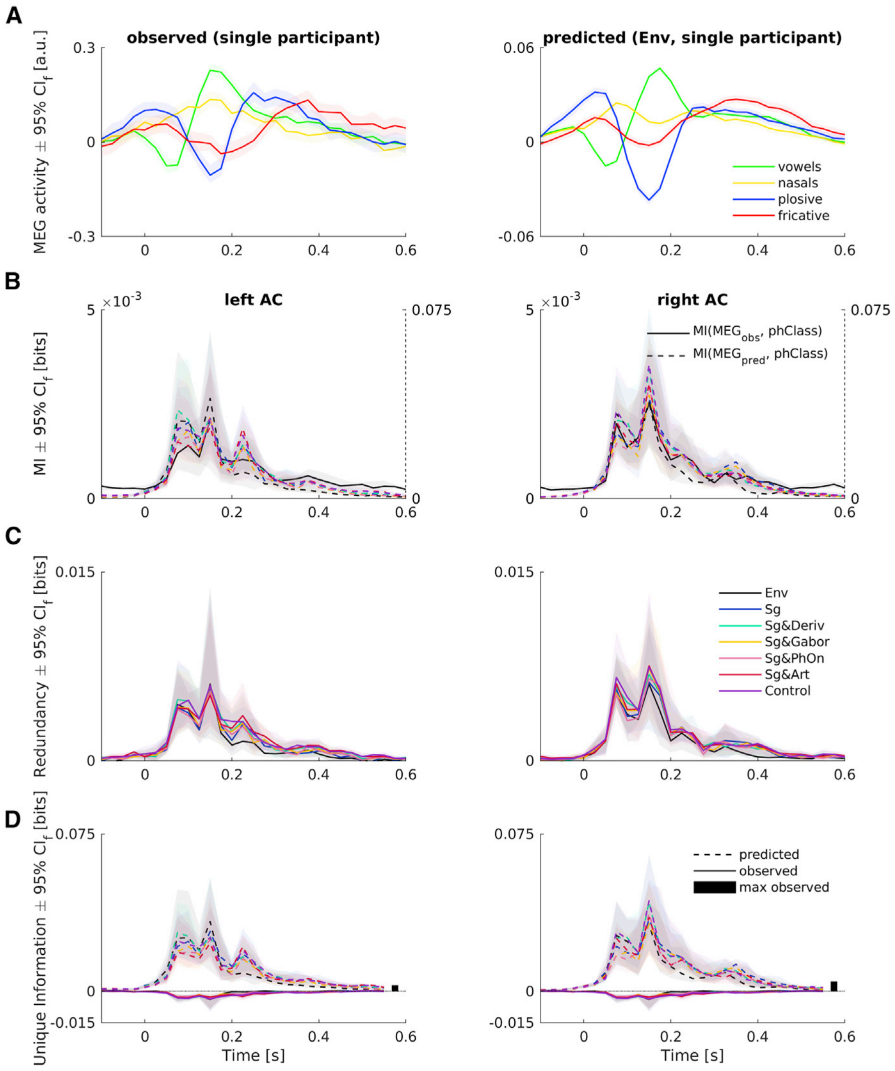

Figure 5. Phoneme-Related Fields Captured by Model Predictions

(A) Phoneme-related fields of a single participant in (left) observed and (right) predicted MEG (from Env feature space). Colors code for four different phoneme classes that represent four manners of articulation.

(B) MI of observed (solid lines, left y axes) and predicted MEG (dashed and colored, right y axes) about the four phoneme categories in the left and right ACs. Color coding of feature spaces is the same as in (C).

(C) Redundancy from PID (amount of information that observed and predicted MEG share about the four manners of articulation). Shown are medians across all participants ± 95% (frequentist) confidence intervals (CI_f), bootstrapped with 10,000 samples.

(D) Unique information of observed (solid) and predicted MEG about the manners of articulation. Maximum information uniquely available from observed MEG across all participants, feature spaces, and time points are shown as black bars. Color coding of feature spaces is the same as in (C). Shown are medians across all participants ± 95% (frequentist) CI_f, bootstrapped with 10,000 samples.

See also Figure S4.

To assess the amount of information that is shared by the observed and predicted time series about these manners of articulation, and the amount of information that is unique to them, we performed PIDs with observed and predicted time series as sources and the manners of articulation as targets. This analysis should reveal whether the observed MEG contained information about these manners of articulation that is different from that obtained, for example, from the speech envelope when convolved with an encoding model temporal response function (TRF).

The PIDs resulted in profiles of redundancy that closely resembled the marginal MI profiles for both hemispheres and for all feature spaces alike (Figure 5C). Most importantly, the information that was unique to the predicted MEG exhibited the same patterns (Figure 5D, dashed lines), while the information unique to the observed MEG (solid lines) was negative; i.e., this information represented misinformation with respect to the predicted MEG source. This means that there were trials where an observer predicting phoneme classes optimally from the observed MEG would make a mistake (hence misinformation)

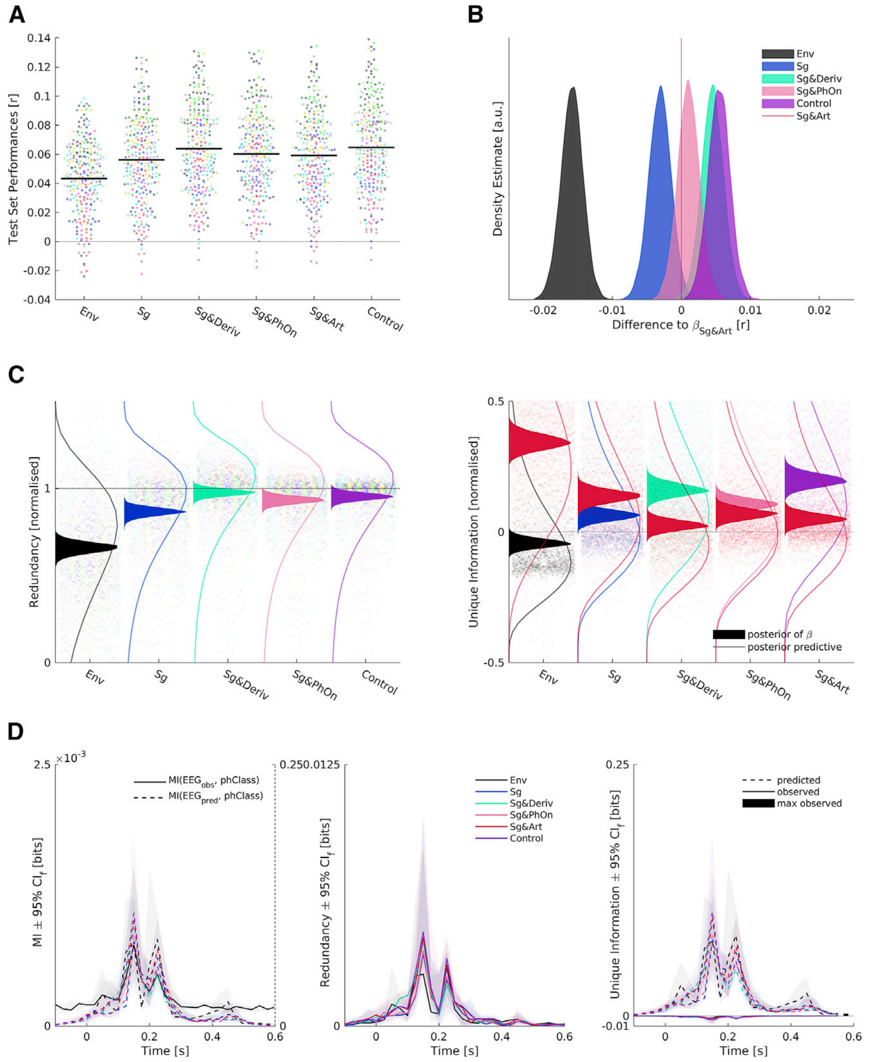

Figure 6. Analysis of EEG Data

(A) Test set performances of forward models. Left: each dot shows the performance in one test set averaged across electrodes. Colors code individual participants (n = 13), and black lines show pooled medians.

(B) Samples from posterior distribution of differences of beta estimates of competing feature spaces and the benchmark Sg & Art feature space. Colors code feature spaces.

(C) PID results normalized by MI of predictions based on Sg & Art features and observed EEG signals. Each dot is one test set prediction of one participant and electrode. Samples from posterior distributions of effects of feature spaces are overlaid as filled areas, and posterior predictive distributions are shown as lines. (Left) Redundancy of predictions based on benchmark articulatory features and competing feature spaces about observed EEG signals. Dot colors represent a participant; filled area and line colors represent feature spaces. (Right) Unique information of benchmark articulatory features (red) and competing feature spaces about observed EEG signals. Colors of dots, filled areas, and lines represent feature spaces.

(D) Phoneme related potential analysis. Colors represent feature spaces, and shaded areas denote 95% (frequentist) confidence intervals (CI_f), bootstrapped with 10,000 samples. All traces show averages across participants and electrodes. (Left) MI of observed (solid black line, left y axis of subplot) and predicted (dashed colored lines, right y axis of subplot) EEG about four manner of articulation phoneme classes ("phClass"). (Middle) Redundancy—information shared by observed and predicted EEG from different feature spaces about phoneme classes. (Right) Unique information of observed (solid lines) and predicted (dashed lines) EEG about phoneme classes. Maximum of information uniquely available from observed EEG across all participants, feature spaces, and time points, shown as black bar.

See also Figures S5 and S6.

that an observer of the predicted MEG would not make (hence unique to observed MEG; see STAR Methods for more details on negative unique information). Thus, there was no relevant information about these manners of articulation present in the observed MEG that could not be retrieved from responses modeled with a convolution of any of our feature spaces with an encoding model filter. This pattern of results was also essentially the same for both hemispheres and for all feature spaces.

Taken together, these results demonstrate that models based on all of our feature spaces could fully account for the information about these four manners of articulation that was decodable from the observed MEG responses.

Replication Using a Publicly Available EEG Dataset

The original report of the effect of a performance gain provided by articulatory features over spectrograms alone was derived from EEG data [10]. Since MEG and EEG are sensitive to different sources [48], it is possible that the MEG sensors we used here were blind to parts of the effect. We therefore investigated whether we could replicate our MEG results using EEG data. We analyzed 13 participants for whom data with 128 channel recordings of approximately an hour are publicly available [20, 49]. On the stimulus side, we used the same analysis pipeline as for the MEG dataset. However, due to the higher noise level of the EEG data [47], we did not try to fit the high-dimensional Gabor feature space. Instead, we concentrated on comparing the benchmark articulatory feature space to the lower dimensional acoustic feature spaces that had best explained the MEG data. We fitted cross-validated encoding models to the scalp-level EEG data and focused our modeling on the 12 electrodes reported in the original publication [10].

Using the same Bayesian modeling approach, results derived from the EEG data closely accorded with those derived from the MEG data (Figure 6B). Our analysis replicated the gain in performance of the benchmark articulatory feature space compared to spectrograms alone ($\Delta = 0.0031$; $f_{h_1} = 0.9712$). We again found that the benchmark articulatory feature space was outperformed by the combination of spectrograms and their

rectified temporal derivatives ($\Delta = 0.0045$; $f_{h_1} = 0.9963$). Also, we again found that combining the articulatory features with the best acoustic features only led to a negligible increase in performance ($\Delta = 0.0009$; $f_{h_1} = 0.7218$). In addition, as before, the benchmark articulatory feature space performance was not stronger than that of the spectrograms and phoneme onsets combined ($\Delta = 0.0010$; $f_{h_1} = 0.2577$). Lastly, we found that all competing feature spaces outperformed the one-dimensional envelope ($\Delta = 0.0128$–0.0213, all $f_{h_1} = 1$). These results thus show that—in terms of prediction performance—acoustic features outperform the more complex articulatory features, which perform on a par with features that only describe the phoneme timing.

Note that when we replaced the log-mel spectrogram features chosen in the present study with a spectrogram more closely modeled after the one used in [10], we obtained generally lower performances and also a different pattern of results. Crucially, we found that this could be attributed to a compressive non-linearity as included in the log-mel spectrogram (see Figure S6 for a more detailed explanation). Taken together, these results further support the notion that simple and physiologically motivated transformations of the auditory stimulus can make important differences to the interpretation of more-complex annotated features.

Next, we considered the results of a PID analysis that assessed the degree to which the predictions of competing feature spaces shared information about the observed EEG responses with that of the benchmark feature space and the degree to which they contributed unique information (Figure 6C). We again found that the predictions based on the best acoustic feature space were highly redundant with predictions based on the benchmark articulatory features (mean of the corresponding effect = 0.9776; 95% CI = 0.9358–1.0167). The same was the case for the combination of the best acoustic features and the articulatory features (mean = 0.9527; 95% CI = 0.9104–0.9945). We also again found that the unique information contributed by the benchmark articulatory features was close to 0 (mean of the corresponding effect = 0.0231; 95% CI = −0.0115 to 0.0657), while the unique information contributed by the best acoustic feature space was weakly positive (mean of the corresponding effect = 0.1575; 95% CI = 0.1121–0.2109). Lastly, the amount of information only available when jointly considering the best acoustic features and the benchmark articulatory features (i.e., the synergy) was an order of magnitude lower than that of the redundancy and did not exceed noise thresholds (see Figure S5), which agreed with the finding that combining the best acoustic features and the articulatory features did not lead to an improvement over the best acoustic features.

Similar to our results using MEG, the combination of spectrograms and phoneme onsets produced slightly lower levels of redundancy compared to the best acoustic model (mean = 0.9317; 95% CI = 0.8902–0.9765), and even lower levels of redundancy were obtained for spectrograms alone (mean = 0.8658; 95% CI = 0.8157–0.9118), and for the envelope (mean = 0.6714; 95% CI = 0.5956–0.7205).

Based on these results, we concluded that in both MEG and EEG data, the increased performance provided by benchmark articulatory features over spectrograms alone could be explained by a combination of spectrograms and their rectified temporal derivatives. This purely acoustic feature space achieved higher overall performance in predicting EEG responses. It did so by explaining the same information as the benchmark articulatory features. However, it also carried information that was not available from the predictions based on the benchmark articulatory features.

Finally, we also found a very similar pattern of results in an analysis of phoneme-evoked responses (Figure 6C). The MI of the observed EEG time series and the four phoneme classes was mostly shared with that of the predicted time series based on all feature spaces. The predicted time series could thus account for a substantial amount of positive unique information, while the observed EEG time series could only contribute negative unique information, i.e., misinformation. The observed EEG responses thus did not contain any more information about the manners of articulation than did the EEG response predictions based solely on the envelope.

DISCUSSION

In this study, we set out to investigate to which degree signatures of "pre-lexical abstraction" in MEEG responses to speech can be explained with simpler, purely acoustic models. Our results suggest that care must be taken when interpreting the results of encoding or decoding models that consider higher-order constructs, such as the articulatory features of phonemes. We showed that the predictive information that can be derived from articulatory features is rooted in the timing information of these features rather than in a more-detailed characterization of the phoneme. Similarly, the ability to reliably decode subgroups of phonemes from MEEG data can be explained by our simplest feature model, i.e., it is a direct consequence of MEEG speech envelope tracking. It should therefore not be interpreted as evidence of more complex speech processing being reflected in the recorded signal. Based on these results, we argue here for the consideration of algorithmically interpretable and physiologically plausible models of sensory encoding, for which annotated feature spaces can nevertheless serve as excellent benchmarks.

An inevitable limitation of this study is that our results cannot ultimately prove the absence of explanatory power unique to the articulatory features. It is possible that analysis pipelines exist that could carve out parts of the responses such that the articulatory features could beat our best acoustic feature space. However, in our analyses, the articulatory features were given strong chances to predict response variance. And we could indeed replicate the originally reported effect of a performance gain over spectrograms alone, only to then find a more parsimonious explanation for this gain. Moreover, our findings suggest that if the articulatory features could better explain certain parts of the responses, these parts would account for a relatively small portion of the total response variance. Given the already small effect sizes, it would then be possible that additional and similarly simple transformations of the acoustics could compensate for possible articulatory advantages. The same holds true for recent demonstrations of more-sophisticated linguistic feature spaces [18, 20]. Essentially, this line of reasoning thus drives home our main point. Any invocation of exciting, high-level feature spaces will always entail the heavy burden of proof of the absence of simpler explanations [56]. This should by no

means discourage inspiring investigations from using such high-level feature spaces, but it should encourage researchers to nevertheless continue to consider simpler explanations.

Similarly, the ability to decode high-level semantic or phonetic properties of speech from evoked neural data tantalizingly suggests that the measured neural response reflects high-level processing. However, in general it is extremely difficult to control properly for all possible low-level stimulus properties, which could confound the interpretation of the high-level feature decoding. Applying decoding analyses to the predictions of forward models as we suggest here provides one way to address this issue. If, as we find here, the high-level feature can indeed be decoded from the prediction of a forward model based on low-level stimulus features, it suggests that the decoding results should not be interpreted as strong evidence of high-level neural processing.

We used in our study a source reconstruction approach that used data derived from two sources in bilateral auditory cortices. Source-level MEG data in Brodbeck et al. [18], for example, suggest that multiple, superior temporal sources related to speech processing are robustly separable. This could be explained by the difference in source reconstruction algorithms. Given the relatively coarse, spatial resolution of our source-level data, we chose not to focus our analysis on modeling activity reconstructed from multiple locations in source space. Instead, we invested our computational resources in a detailed analysis of responses from a single point per hemisphere that accounted for much of the speech-related variance. This allowed us to flexibly optimize analysis parameters specific to participants, hemispheres, and feature (sub-)spaces. We believe that this data-driven approach to parameter settings [57] marks an important step toward more-principled pipelines in neuroimaging [58], and our approach was inspired by growing efforts to avoid MEG analysis parameter settings based on tradition [59, 60].

Since forward-encoding models promise to inform theories of neuronal computations, what are the potential implications of this study? The central question of interest concerns the origins of the response variance that is commonly explained by the best acoustic and articulatory benchmark features. However, interpreting the results of encoding and decoding models with regard to such a causal question is never trivial [61, 62]. The feature spaces considered here reflect functional—not mechanistic—models [63] of varying predictive performance. What they essentially relate is the input of the waveform of a speech stimulus to the output of MEEG responses. These responses are far from reflecting the entire, drastically higher-dimensional cortical auditory representation of the stimulus. It seems safe to conclude that this part of brain activity cannot readily provide a window to arbitrary high-level cognitive processes.

Furthermore, an algorithmic consideration of our best acoustic feature space rather points to operations that occur relatively early in auditory processing. A spectral decomposition of compressed dynamic range is typically part of cochlear models [3, 53]. An additional temporal derivative and half-wave rectification might possibly be implemented by the various stations along the subcortical auditory pathway. The question then is why cortical neuronal mass signals [64] are time-locked to this result of very early auditory processing, and whether these low-frequency cortical responses carry such information so that further cortical processes react to it. Deeper insights into this problem will also have to consider proxies to what downstream neurons are encoding, such as the final behavioral responses [65–71].

Despite these caveats, it is interesting to speculate how the feature spaces considered here might reflect aspects of actual neuronal computations. Unlike modern ASR systems that can, with limitations, understand a speaker's intention [72], the mid-level feature spaces considered here are all far from this feat. Nevertheless, they can be interpreted as contributing to this goal. The information bottleneck framework [73], for example, suggests that feature spaces should allow information compression, i.e., gradual decreasing stimulus fidelity, while retaining relevant aspects of the input. The log-mel spectrograms allow us to discard irrelevant spectral and dynamic ranges, and Gabor-filtering can do the same for spectrotemporal patterns relevant for ASR systems [28]. This decomposition seems to be especially beneficial for speech in noise, when features similar to the best acoustic feature space used here can be used to exploit the rapid amplitude dynamics in speech signals to the benefit of ASR systems [74]. It is thus conceivable that the predictive performance of this feature space could be rooted in a tuning of the auditory system to ubiquitous noisy listening environments. Hypotheses about the processing of speech in noise are, however, best examined in datasets that sample the stimulus space correspondingly [42, 75].

Another interesting observation is that the edges of these rapid amplitude dynamics coincide with transitions to the central vowels of syllables [40]. A rich literature is available on the interpretation of low-frequency signals as a signature of a chunking of the speech signal into syllable-like units [36–38, 76–79]. An eventual goal would, however, be to treat mid-level representations as less independent from the more-abstract aspects of speech understanding. Extracting the intermediate representations generated while embedding speech into fixed dimensional semantic vectors [80] could be a promising step toward an unbiased and context dependent description of speech signals.

Conclusion

In a data-driven approach, we have studied models that explain cortical neuronal responses as captured by source-localized MEG and sensor level EEG in a story-listening paradigm. Our results underscore that annotated linguistic feature spaces are useful tools to explore neuronal responses to speech and serve as excellent benchmarks. We find their performance for explaining neuronal responses of high temporal resolution to be exceeded and explained by a simple low-level acoustic feature space that capitalizes on spectrotemporal dynamics. Thus, we conclude that the consideration of parsimonious, algorithmically interpretable and physiologically plausible features will eventually lead to clearer explanations of observed neuronal responses.

STAR★METHODS

Detailed methods are provided in the online version of this paper and include the following:

- KEY RESOURCES TABLE
- CONTACT FOR REAGENT AND RESOURCE SHARING

- EXPERIMENTAL METHODS AND SUBJECT DETAILS
  - Participants
- METHOD DETAILS
  - MEG recording, preprocessing, and spatial filtering
- QUANTIFICATION AND STATISTICAL ANALYSIS
  - Model comparisons
- DATA AND SOFTWARE AVAILABILITY

SUPPLEMENTAL INFORMATION

Supplemental Information can be found online at https://doi.org/10.1016/j.cub.2019.04.067.

ACKNOWLEDGMENTS

C.D. is funded by the College of Science and Engineering at the University of Glasgow; R.A.A.I. (UK; 214120/Z/18/Z) and J.G. (UK; 098433) received support from the Wellcome Trust. We thank Moritz Boos, Jan-Mathijs Schoffelen, Dale J. Barr, and Christoph Scheepers for helpful discussions as well as Giovanni M. Di Liberto and Edmund C. Lalor for sharing their data, useful discussions, and insightful debates.

AUTHOR CONTRIBUTIONS

C.D., R.A.A.I., and J.G. conceived of and designed the experiment. C.D. collected and analyzed the data. C.D. and R.A.A.I. contributed analytic tools. C.D., R.A.A.I., and J.G. wrote the paper. J.G. acquired the financial support for the project leading to this manuscript.

DECLARATION OF INTERESTS

The authors declare no competing interests.

Received: October 22, 2018
Revised: March 25, 2019
Accepted: April 25, 2019
Published: May 23, 2019

REFERENCES

1. Pisoni, D.B., and Luce, P.A. (1987). Acoustic-phonetic representations in word recognition. Cognition *25*, 21–52.
2. DeWitt, I., and Rauschecker, J.P. (2012). Phoneme and word recognition in the auditory ventral stream. Proc. Natl. Acad. Sci. USA *109*, E505–E514.
3. Verhulst, S., Altoè, A., and Vasilkov, V. (2018). Computational modeling of the human auditory periphery: auditory-nerve responses, evoked potentials and hearing loss. Hear. Res. *360*, 55–75.
4. Sitek, K.R., Gulban, O.F., Calabrese, E., Johnson, G.A., Ghosh, S.S., and Martino, F.D. (2019). Mapping the human subcortical auditory system using histology, post mortem MRI and in vivo MRI at 7T. bioRxiv. https://doi.org/10.1101/568139.
5. Młynarski, W., and McDermott, J.H. (2018). Learning midlevel auditory codes from natural sound statistics. Neural Comput. *30*, 631–669.
6. Naselaris, T., Kay, K.N., Nishimoto, S., and Gallant, J.L. (2011). Encoding and decoding in fMRI. Neuroimage *56*, 400–410.
7. Holdgraf, C.R., Rieger, J.W., Micheli, C., Martin, S., Knight, R.T., and Theunissen, F.E. (2017). Encoding and decoding models in cognitive electrophysiology. Front. Syst. Neurosci. *11*, 61.
8. Theunissen, F.E., and Elie, J.E. (2014). Neural processing of natural sounds. Nat. Rev. Neurosci. *15*, 355–366.
9. Hamilton, L.S., and Huth, A.G. (2018). The revolution will not be controlled: natural stimuli in speech neuroscience. Lang. Cogn. Neurosci. Published online July 22, 2018. https://doi.org/10.1080/23273798.2018.1499946.
10. Di Liberto, G.M., O'Sullivan, J.A., and Lalor, E.C. (2015). Low-frequency cortical entrainment to speech reflects phoneme-level processing. Curr. Biol. *25*, 2457–2465.
11. Huth, A.G., de Heer, W.A., Griffiths, T.L., Theunissen, F.E., and Gallant, J.L. (2016). Natural speech reveals the semantic maps that tile human cerebral cortex. Nature *532*, 453–458.
12. de Heer, W.A., Huth, A.G., Griffiths, T.L., Gallant, J.L., and Theunissen, F.E. (2017). The hierarchical cortical organization of human speech processing. J. Neurosci. *37*, 6539–6557.
13. Berezutskaya, J., Freudenburg, Z.V., Güçlü, U., van Gerven, M.A.J., and Ramsey, N.F. (2017). Neural tuning to low-level features of speech throughout the perisylvian cortex. J. Neurosci. *37*, 7906–7920.
14. Forte, A.E., Etard, O., and Reichenbach, T. (2017). The human auditory brainstem response to running speech reveals a subcortical mechanism for selective attention. eLife *6*, e27203.
15. Maddox, R.K., and Lee, A.K.C. (2018). Auditory brainstem responses to continuous natural speech in human listeners. eNeuro *5*, ENEURO.0441-17.2018.
16. Kell, A.J.E., Yamins, D.L.K., Shook, E.N., Norman-Haignere, S.V., and McDermott, J.H. (2018). A task-optimized neural network replicates human auditory behavior, predicts brain responses, and reveals a cortical processing hierarchy. Neuron *98*, 630–644.e16.
17. Brodbeck, C., Presacco, A., and Simon, J.Z. (2018). Neural source dynamics of brain responses to continuous stimuli: speech processing from acoustics to comprehension. Neuroimage *172*, 162–174.
18. Brodbeck, C., Hong, L.E., and Simon, J.Z. (2018). Rapid transformation from auditory to linguistic representations of continuous speech. Curr. Biol. *28*, 3976–3983.
19. Biesmans, W., Das, N., Francart, T., and Bertrand, A. (2017). Auditory-inspired speech envelope extraction methods for improved EEG-based auditory attention detection in a cocktail party scenario. IEEE Trans. Neural Syst. Rehabil. Eng. *25*, 402–412.
20. Broderick, M.P., Anderson, A.J., Di Liberto, G.M., Crosse, M.J., and Lalor, E.C. (2018). Electrophysiological correlates of semantic dissimilarity reflect the comprehension of natural, narrative speech. Curr. Biol. *28*, 803–809.
21. Mesgarani, N., Cheung, C., Johnson, K., and Chang, E.F. (2014). Phonetic feature encoding in human superior temporal gyrus. Science *343*, 1006–1010.
22. Khalighinejad, B., Cruzatto da Silva, G., and Mesgarani, N. (2017). Dynamic encoding of acoustic features in neural responses to continuous speech. J. Neurosci. *37*, 2176–2185.
23. Obleser, J., and Eisner, F. (2009). Pre-lexical abstraction of speech in the auditory cortex. Trends Cogn. Sci. *13*, 14–19.
24. Hasson, U., Egidi, G., Marelli, M., and Willems, R.M. (2018). Grounding the neurobiology of language in first principles: The necessity of non-language-centric explanations for language comprehension. Cognition *180*, 135–157.
25. Massaro, D.W. (1974). Perceptual units in speech recognition. J. Exp. Psychol. *102*, 199–208.
26. Lotto, A.J., and Holt, L.L. (2000). The illusion of the phoneme. In The Panels, S.J. Billings, ed. (Chicago Linguistic Society), pp. 191–204.
27. Kriegeskorte, N., and Douglas, P.K. (2018). Cognitive computational neuroscience. Nat. Neurosci. *21*, 1148–1160.
28. Schädler, M., Meyer, B.T., and Kollmeier, B. (2012). Spectro-temporal modulation subspace-spanning filter bank features for robust automatic speech recognition. J. Acoust. Soc. Am. *131*, 4134–4151.
29. Qiu, A., Schreiner, C.E., and Escabí, M.A. (2003). Gabor analysis of auditory midbrain receptive fields: spectro-temporal and binaural composition. J. Neurophysiol. *90*, 456–476.
30. Pasley, B.N., David, S.V., Mesgarani, N., Flinker, A., Shamma, S.A., Crone, N.E., Knight, R.T., and Chang, E.F. (2012). Reconstructing speech from human auditory cortex. PLoS Biol. *10*, e1001251.

31. Santoro, R., Moerel, M., De Martino, F., Goebel, R., Ugurbil, K., Yacoub, E., and Formisano, E. (2014). Encoding of natural sounds at multiple spectral and temporal resolutions in the human auditory cortex. PLoS Comput. Biol. *10*, e1003412.
32. Santoro, R., Moerel, M., De Martino, F., Valente, G., Ugurbil, K., Yacoub, E., and Formisano, E. (2017). Reconstructing the spectrotemporal modulations of real-life sounds from fMRI response patterns. Proc. Natl. Acad. Sci. USA *114*, 4799–4804.
33. Norman-Haignere, S.V., and McDermott, J.H. (2018). Neural responses to natural and model-matched stimuli reveal distinct computations in primary and nonprimary auditory cortex. PLoS Biol. *16*, e2005127.
34. Schönwiesner, M., and Zatorre, R.J. (2009). Spectro-temporal modulation transfer function of single voxels in the human auditory cortex measured with high-resolution fMRI. Proc. Natl. Acad. Sci. USA *106*, 14611–14616.
35. Prendergast, G., Johnson, S.R., and Green, G.G.R. (2010). Temporal dynamics of sinusoidal and non-sinusoidal amplitude modulation. Eur. J. Neurosci. *32*, 1599–1607.
36. Hertrich, I., Dietrich, S., Trouvain, J., Moos, A., and Ackermann, H. (2012). Magnetic brain activity phase-locked to the envelope, the syllable onsets, and the fundamental frequency of a perceived speech signal. Psychophysiology *49*, 322–334.
37. Gross, J., Hoogenboom, N., Thut, G., Schyns, P., Panzeri, S., Belin, P., and Garrod, S. (2013). Speech rhythms and multiplexed oscillatory sensory coding in the human brain. PLoS Biol. *11*, e1001752.
38. Doelling, K.B., Arnal, L.H., Ghitza, O., and Poeppel, D. (2014). Acoustic landmarks drive delta-theta oscillations to enable speech comprehension by facilitating perceptual parsing. Neuroimage *85*, 761–768.
39. Hamilton, L.S., Edwards, E., and Chang, E.F. (2018). A spatial map of onset and sustained responses to speech in the human superior temporal gyrus. Curr. Biol. *28*, 1860–1871.e4.
40. Oganian, Y., and Chang, E.F. (2018). A speech envelope landmark for syllable encoding in human superior temporal gyrus. bioRxiv. https://doi.org/10.1101/388280.
41. Hambrook, D.A., and Tata, M.S. (2014). Theta-band phase tracking in the two-talker problem. Brain Lang. *135*, 52–56.
42. Fiedler, L., Wöstmann, M., Herbst, S.K., and Obleser, J. (2019). Late cortical tracking of ignored speech facilitates neural selectivity in acoustically challenging conditions. Neuroimage *186*, 33–42.
43. Ince, R.A.A., Giordano, B.L., Kayser, C., Rousselet, G.A., Gross, J., and Schyns, P.G. (2017). A statistical framework for neuroimaging data analysis based on mutual information estimated via a gaussian copula. Hum. Brain Mapp. *38*, 1541–1573.
44. Varoquaux, G., Raamana, P.R., Engemann, D.A., Hoyos-Idrobo, A., Schwartz, Y., and Thirion, B. (2017). Assessing and tuning brain decoders: cross-validation, caveats, and guidelines. Neuroimage *145* (Pt B), 166–179.
45. Acerbi, L., and Ma, W.J. (2017). Practical Bayesian optimization for model fitting with Bayesian adaptive direct search. Adv. Neural Inf. Process. Syst. *30*, 1834–1844.
46. Ince, R.A.A. (2017). Measuring multivariate redundant information with pointwise common change in surprisal. Entropy *19*, 21–57.
47. Destoky, F., Philippe, M., Bertels, J., Verhasselt, M., Coquelet, N., Vander Ghinst, M., Wens, V., De Tiège, X., and Bourguignon, M. (2019). Comparing the potential of MEG and EEG to uncover brain tracking of speech temporal envelope. Neuroimage *184*, 201–213.
48. Cohen, D., and Cuffin, B.N. (1983). Demonstration of useful differences between magnetoencephalogram and electroencephalogram. Electroencephalogr. Clin. Neurophysiol. *56*, 38–51.
49. Broderick, M.P., Anderson, A.J., Di Liberto, G.M., Crosse, M.J., and Lalor, E.C. (2018). Data from: electrophysiological correlates of semantic dissimilarity reflect the comprehension of natural, narrative speech. Dryad Digital Repository. Published online February 23, 2018. https://doi.org/10.5061/dryad.070jc/5.
50. Honey, C.J., Thesen, T., Donner, T.H., Silbert, L.J., Carlson, C.E., Devinsky, O., Doyle, W.K., Rubin, N., Heeger, D.J., and Hasson, U. (2012). Slow cortical dynamics and the accumulation of information over long timescales. Neuron *76*, 423–434.
51. Farahibozorg, S.R., Henson, R.N., and Hauk, O. (2018). Adaptive cortical parcellations for source reconstructed EEG/MEG connectomes. Neuroimage *169*, 23–45.
52. Ding, N., and Simon, J.Z. (2012). Emergence of neural encoding of auditory objects while listening to competing speakers. Proc. Natl. Acad. Sci. USA *109*, 11854–11859.
53. Chi, T., Ru, P., and Shamma, S.A. (2005). Multiresolution spectrotemporal analysis of complex sounds. J. Acoust. Soc. Am. *118*, 887–906.
54. Bürkner, P.C. (2017). brms: an R package for Bayesian multilevel models using Stan. J. Stat. Softw. *80*, 1–28.
55. Williams, P.L., and Beer, R.D. (2012). Nonnegative Decomposition of Multivariate Information. arXiv, arXiv:1004.2515. https://arxiv.org/abs/1004.2515.
56. Sassenhagen, J. (2018). How to analyse electrophysiological responses to naturalistic language with time-resolved multiple regression. Lang. Cogn. Neurosci. *34*, 474–490.
57. Hahn, T., Emden, D., Grotegerd, D., Kaehler, C., Leenings, R., and Winter, N. (2018). A Python-based Hyperparameter Optimization Toolbox for Neural Networks designed to accelerate and simplify the construction, training, and evaluation of machine learning models. https://www.photon-ai.com/.
58. Bzdok, D., and Yeo, B.T.T. (2017). Inference in the age of big data: future perspectives on neuroscience. Neuroimage *155*, 549–564.
59. Woolrich, M., Hunt, L., Groves, A., and Barnes, G. (2011). MEG beamforming using Bayesian PCA for adaptive data covariance matrix regularization. Neuroimage *57*, 1466–1479.
60. Engemann, D.A., and Gramfort, A. (2015). Automated model selection in covariance estimation and spatial whitening of MEG and EEG signals. Neuroimage *108*, 328–342.
61. Weichwald, S., Meyer, T., Özdenizci, O., Schölkopf, B., Ball, T., and Grosse-Wentrup, M. (2015). Causal interpretation rules for encoding and decoding models in neuroimaging. Neuroimage *110*, 48–59.
62. Kriegeskorte, N., and Douglas, P.K. (2018). Interpreting Encoding and Decoding Models. arXiv, arXiv:1812.00278. http://arxiv.org/abs/1812.00278.
63. Kay, K.N. (2018). Principles for models of neural information processing. Neuroimage *180* (Pt A), 101–109.
64. Panzeri, S., Macke, J.H., Gross, J., and Kayser, C. (2015). Neural population coding: combining insights from microscopic and mass signals. Trends Cogn. Sci. *19*, 162–172.
65. Williams, M.A., Dang, S., and Kanwisher, N.G. (2007). Only some spatial patterns of fMRI response are read out in task performance. Nat. Neurosci. *10*, 685–686.
66. de-Wit, L., Alexander, D., Ekroll, V., and Wagemans, J. (2016). Is neuroimaging measuring information in the brain? Psychon. Bull. Rev. *23*, 1415–1428.
67. Panzeri, S., Harvey, C.D., Piasini, E., Latham, P.E., and Fellin, T. (2017). Cracking the neural code for sensory perception by combining statistics, intervention, and behavior. Neuron *93*, 491–507.
68. Bouton, S., Chambon, V., Tyrand, R., Guggisberg, A.G., Seeck, M., Karkar, S., van de Ville, D., and Giraud, A.-L. (2018). Focal versus distributed temporal cortex activity for speech sound category assignment. Proc. Natl. Acad. Sci. USA *115*, E1299–E1308.
69. Keitel, A., Gross, J., and Kayser, C. (2018). Perceptually relevant speech tracking in auditory and motor cortex reflects distinct linguistic features. PLoS Biol. *16*, e2004473.
70. Carlson, T., Goddard, E., Kaplan, D.M., Klein, C., and Ritchie, J.B. (2018). Ghosts in machine learning for cognitive neuroscience: Moving from data to theory. Neuroimage *180* (Pt A), 88–100.

71. Brette, R. (2018). Is coding a relevant metaphor for the brain? bioRxiv. https://doi.org/10.1101/168237.

72. Sarikaya, R., Crook, P.A., Marin, A., Jeong, M., Robichaud, J.P., Celikyilmaz, A., Kim, Y.B., Rochette, A., Khan, O.Z., Liu, X., et al. (2016). An overview of end-to-end language understanding and dialog management for personal digital assistants. http://ieeexplore.ieee.org/document/7846294/.

73. Tishby, N., Pereira, F.C., and Bialek, W. (2000). The information bottleneck method. arXiv, arXiv:physics/0004057. http://arxiv.org/abs/physics/0004057.

74. Kumar, K., Kanwoo, C.J., and Stern, R.M. (2011). Delta-spectral-cepstral coefficients for robust speech recognition. International Conference on Acoustics, Speech and Signal Processing (ICASSP), pp. 4784–4787.

75. Giordano, B.L., Ince, R.A.A., Gross, J., Schyns, P.G., Panzeri, S., and Kayser, C. (2017). Contributions of local speech encoding and functional connectivity to audio-visual speech perception. eLife *6*, e24763.

76. Hyafil, A., Fontolan, L., Kabdebon, C., Gutkin, B., and Giraud, A.-L. (2015). Speech encoding by coupled cortical theta and gamma oscillations. eLife *4*, e06213.

77. Räsänen, O., Doyle, G., and Frank, M.C. (2018). Pre-linguistic segmentation of speech into syllable-like units. Cognition *171*, 130–150.

78. Giraud, A.L., and Poeppel, D. (2012). Cortical oscillations and speech processing: emerging computational principles and operations. Nat. Neurosci. *15*, 511–517.

79. Ghitza, O. (2013). The theta-syllable: a unit of speech information defined by cortical function. Front. Psychology *4*, 138.

80. Chung, Y.A., Weng, W.H., Tong, S., and Glass, J. (2018). Unsupervised Cross-Modal Alignment of Speech and Text Embedding Spaces. arXiv, arXiv:1805.07467. http://arxiv.org/abs/1805.07467.

81. R Core Team (2013). R: A Language and Environment for Statistical Computing (R Foundation for Statistical Computing). http://www.R-project.org/.

82. Brainard, D.H. (1997). The psychophysics toolbox. Spat. Vis. *10*, 433–436.

83. Oostenveld, R., Fries, P., Maris, E., and Schoffelen, J.-M. (2011). FieldTrip: Open source software for advanced analysis of MEG, EEG, and invasive electrophysiological data. Comput. Intell. Neurosci. *2011*, 156869.

84. Abraham, A., Pedregosa, F., Eickenberg, M., Gervais, P., Mueller, A., Kossaifi, J., Gramfort, A., Thirion, B., and Varoquaux, G. (2014). Machine learning for neuroimaging with scikit-learn. Front. Neuroinformatics *8*, 14.

85. Boersma, P. (2001). Praat, a system for doing phonetics by computer. Glot Int. *5*, 341–345.

86. Yuan, J., and Liberman, A.M. (2008). Speaker identification on the SCOTUS corpus. J. Acoust. Soc. Am. *123*, 3878.

87. Nolte, G. (2003). The magnetic lead field theorem in the quasi-static approximation and its use for magnetoencephalography forward calculation in realistic volume conductors. Phys. Med. Biol. *48*, 3637–3652.

88. Van Veen, B.D., van Drongelen, W., Yuchtman, M., and Suzuki, A. (1997). Localization of brain electrical activity via linearly constrained minimum variance spatial filtering. IEEE Trans. Biomed. Eng. *44*, 867–880.

89. Crosse, M.J., DiLiberto, G.M., Bednar, A., and Lalor, E.C. (2016). The Multivariate Temporal Response Function (mTRF) Toolbox: a MATLAB toolbox for relating neural signals to continuous stimuli. Front. Hum. Neurosci. *10*, 604.

90. Stan Development Team (2018). RStan: the R interface to Stan. http://mc-stan.org/.

91. Wibral, M., Lizier, J.T., and Priesemann, V. (2015). Bits from brains for biologically inspired computing. Front. Robot AI. Published March 19, 2015. https://doi.org/10.3389/frobt.2015.00005.

92. McGill, W.J. (1954). Multivariate information transmission. Psychometrika *19*, 97–116.

93. Park, H., Ince, R.A.A., Schyns, P.G., Thut, G., and Gross, J. (2018). Representational interactions during audiovisual speech entrainment: Redundancy in left posterior superior temporal gyrus and synergy in left motor cortex. PLoS Biol. *16*, e2006558.

94. Ince, R.A.A. (2017). The partial entropy decomposition: decomposing multivariate entropy and mutual information via pointwise common surprisal. arXiv, arXiv:1702.01591. https://arxiv.org/abs/1702.01591.

STAR★METHODS

KEY RESOURCES TABLE

REAGENT or RESOURCE	SOURCE	IDENTIFIER
Deposited Data		
Raw MEG and MRI data	This paper	Available upon request
Stimulus of MEG experiment	Jenkins D.W.; librivox.org	https://ia600309.us.archive.org/7/items/tales_jazz_age_1209_librivox/talesfromjazzage_08_fitzgerald.mp3
EEG data	[49]	https://doi.org/10.17632/pjnkwwzn9x.1
Stimulus of EEG dataset	[49]	Available upon request
Software and Algorithms		
MATLAB R2016a	The Mathworks	RRID: SCR_001622
Python 2.7	Anaconda	https://www.anaconda.com
R	[81]	RRID: SCR_001905
PsychToolBox	[82]	RRID: SCR_002881
FieldTrip	[83]	RRID: SCR_004849
BADS	[45]	https://github.com/lacerbi/bads
gcmi	[43]	https://github.com/robince/gcmi
I_{ccs} PID	[46]	https://github.com/robince/partial-info-decomp
brms	[54]	https://github.com/paul-buerkner/brms
NiLearn	[84]	RRID: SCR_001362
GBFB toolbox	[28]	https://github.com/m-r-s/reference-feature-extraction
Praat	[85]	RRID: SCR_016564
Penn Phonetics Lab Forced Aligner	[86]	http://web.sas.upenn.edu/phonetics-lab/facilities/

CONTACT FOR REAGENT AND RESOURCE SHARING

Further information and requests for resources should be directed to and will be fulfilled by the Lead Contact, Christoph Daube (christoph.daube@gmail.com).

EXPERIMENTAL METHODS AND SUBJECT DETAILS

Participants

24 healthy young participants (native speakers of English, 12 female, mean age 24.0 years, age range 18 – 35 years) agreed to take part in our experiment. They provided informed written consent and received a monetary compensation of £9 per hour. The study was approved by the College of Science and Engineering Ethics Committee at the University of Glasgow (application number: 300170024).

METHOD DETAILS

MEG recording, preprocessing, and spatial filtering

MEG recording

Participants listened to a narrative of 55 minutes duration (“The Curious Case of Benjamin Button,” public domain recording by Don W. Jenkins, librivox.org) while their brain activity was recorded with a 248 channel magnetometer MEG system (MAGNES 3600 WH, 4D Neuroimaging) at a sampling rate of 1017.25 Hz (first 10 participants) and 2034.51 Hz (last 14 participants). Prior to recording, we digitized each participant’s headshape and attached five head position measurement coils to the left and right pre-auricular points as well as to three positions spread across the forehead. The session was split into 6 blocks of equal duration and additionally included a repetition of the last block. The last ten seconds of each block were repeated as a lead-in to the following block to allow listeners to pick up the story. Prior to and after each block, we measured the positions of the coils. If the movement of any of them exceeded 5 mm, we repeated the block. Playback of the story and trigger handling was done using PsychToolBox [82], and sound was

delivered via two MEG compatible Etymotic ER-30 insert earphones. After the recording, participants had to answer 18 multiple choice questions with 3 options each, where the number of correct options could vary between 1 and 3 per question. The questions referred to the entire story, covering three details per recording block. The average performance was 0.95 with a standard deviation of 0.05 and a range from 0.78 to 1.00.

MEG preprocessing

Most of our analyses were carried out within the MATLAB computing environment (v2016a, MathWorks, Natick, MA, USA) using several open-source toolboxes and custom code. Deviations from this are highlighted. Preprocessing was done using the fieldTrip toolbox [83]. Initially, we epoched the data according to the onsets of the full blocks including the ten seconds of lead-in. For noise cancellation, we subtracted the projection of the raw data on an orthogonal basis of the reference channels from the raw data. We manually removed and subsequently replaced artifactual channels with spherical spline interpolations of surrounding channels (mean number of artifactual channels per block: 3.07, standard deviation: 3.64; pooled across participants), replaced squid jumps with DC patches and filtered the signal with a fourth-order forward-reverse zero-phase butterworth high-pass filter with a cutoff-frequency of 0.5 Hz and excluded the lead-in parts from the blocks. We downsampled the data to 125 Hz and found unmixing matrices using the *runica* ICA algorithm. We identified artifactual components reflecting eye or heart activity (mean number of components per block: 6.70, standard deviation: 5.01; pooled across participants). We then unmixed the data at the original sampling rate and back-projected it using mixing matrices where the artifactual components were removed. Finally, we downsampled the data to a sampling rate of 40 Hz.

MEG source space

We employed three different source modeling approaches for our analysis. First, we aimed to identify regions in source space whose activity was in a repeatable relationship with our auditory stimulation (''story-responsive'' regions [12, 50]). Second, we wished to visualize these results on a group-level. Lastly, for our main intention of modeling the story-responsive regions, we designed a framework that would allow us to optimize parameters of our spatial filters as part of a cross-validation, similar to a recent proposal by Engemann & Gramfort [60].

Volume conductor models

For all three approaches, we obtained common volume conductor models. We first aligned individual T1-weighted anatomical MRI scans with the digitized headshapes using the iterative closest point algorithm. Then, we segmented the MRI scans and generated corrected-sphere volume conductor models [87]. We generated grids of points in individual volumes of 5 mm resolution. For group-level visualization purposes, we also generated a grid with 5 mm point spacing in MNI space, and transformed this to individual spaces by applying the inverse of the transform of individual anatomies to MNI space.

Initial data exploration: identification and characterization of story-responsive regions

To identify story-responsive regions in MEG source space, we projected the time-domain sensor level data through rank-reduced linearly constrained minimum variance beamformer spatial filters [88] with the regularization of the sensor covariance matrices λ_{source} set to 5%, using the dipole orientation of maximal power. We correlated the responses to the last block with those to its repeated presentation within each participant to obtain maps of test-retest-R^2. We repeated this using the grids in MNI space we had warped into individual anatomies for a group-level visualization using the *plot_glassbrain* function of the Python module Nilearn [84].

We then explored how many dipoles would explain how much of the repeatable activity in story-responsive regions. It is known that due to the non-uniqueness of the inverse problem, the spatial resolution of MEG source reconstructions is inherently limited. Neighboring grid points are thus often highly correlated, rendering analyses on a full grid highly redundant [51]. To avoid such an unnecessary computational burden for our modeling, we used an information theoretic approach to characterize redundant and unique regions in source space.

First, we computed Mutual Information [MI] [43] at each grid point in individual source spaces between activity in the first and the second repetition, essentially repeating the initial identification of story-responsive regions. Next, we applied the framework of PID [46] to the data of repeated blocks in an iterative approach. PID aims to disentangle redundant, unique and synergistic contributions of two source variables about a target variable (see later section dedicated to PID for more details). As the first source variable, we here used the two-dimensional activity at bilateral grid points of individual peak story-responsivity during the first repetition. We then scanned the whole grid in parallel for both repetitions, using the activity recorded during the first repetition as the second source variable and the activity recorded during the second repetition as the target variable of PIDs (see Video S1 for an intuitive visualization). We were then interested in the resulting maps of redundancy and unique information. The former would allow us to infer to what degree other grid points with high story-responsivity shared their information about the repetition with the grid points of peak story-responsivity. The latter on the other hand would show us where information unexplainable by these two peaks could be found. After this first iteration, we added the grid point of peak unique information to the then three-dimensional first source variable in the PIDs and repeated the computation across the whole grid. We reran this approach for a total of ten iterations. Finally, we computed MI between the two-dimensional activity at bilateral peaks of story-responsivity in the first and the second repetition and compared this to the unique information found in each iteration of our iterative approach.

Optimization of source space coordinates and sensor covariance regularization

In order not to unnecessarily spend computational resources, we wanted to limit our main endeavor of modeling MEG responses to parts of the signal which actually were in a systematic relationship with the stimulus. A straight-forward solution for a selection of these parts would have been to directly use the grid points identified as story-responsive using the test-retest correlation. However,

since it is likely that participants paid a lesser degree of attention to the more predictable repeated presentation of the last chapter, we could not rule out that the test-retest-R^2 maps would be biased toward low-level auditory processing. Furthermore, these maps could be influenced by differences in the position of the participant's head in the scanner as well as the amount of eye blinks and head movements. The peak test-retest points are thus not guaranteed to be the optimal locations for any given feature space model fit, tested over the whole experiment. Moreover, it was possible that different feature spaces would optimally predict distinct regions. Finally, we did not know a-priori what level of regularization of the sensor covariance matrices would be ideal to capture the responses of interest in each individual dataset.

To account for all of these considerations in a data-driven manner, we treated the coordinates of regions of interest as well as the regularization of sensor covariance matrices as hyperparameters of our model, which we optimized by means of a black-box optimization algorithm. We kept the other specifications of the spatial filter design as described above. As initial coordinates, we used the maxima of test-retest-R^2 maps within each hemisphere. The boundaries of the coordinate hyperparameters were defined by the boundaries of the respective hemisphere of the individual brain volume which we shrunk by a factor of 0.99 for this purpose to avoid instabilities of the forward models close to their boundaries (Table S1). In each iteration of the black-box optimization, we then applied a given amount of regularization to the precomputed sensor covariance matrix and computed the leadfield for a given vector $\overline{c} = (X, Y, Z)$ of coordinates in source space using the precomputed volume conductor model for each block. Since the orientation of the resulting dipoles was then arbitrary, i.e., possibly flipped across blocks, we estimated the mean axis of dipoles across blocks and changed the sign of the orientations of dipoles whose dot products with the orientation of the dipole closest to the mean axis were negative. We then recomputed the leadfields for these aligned dipole orientations. Finally, we projected the sensor level data through these spatial filters and z-scored them within each block to account for differences in mean amplitude across blocks.

Stimulus transformations

The speech stimulus was transformed into various feature spaces. We used the GBFB toolbox [28] to obtain 31-channel Log-Mel-Spectrograms (Sg, ranging from 124.1 Hz – 7284.1 Hz) and summed these across the spectral dimension to also obtain the amplitude envelope (Env). Additionally, we filtered the spectrograms with a set of 455 2D Gabor filters ("Gabor") of varying center frequencies corresponding to those of the Sg as well as spectral modulation frequencies Ω (0, 2.9, 6 12.2 and 25 Hz) and temporal modulation frequencies ω (0, 6.2, 9.9, 15.7 and 25 Hz). Notably, this implementation of the toolbox only considers a subset of all possible combinations of center frequency as well as spectral and temporal modulation frequencies to avoid overly redundant features. As a last acoustic feature space, we computed half-wave rectified first derivatives of the individual channels of the spectrograms ("Deriv" [18, 36]).

To construct annotated feature spaces, we used the Penn Phonetics Lab Forced Aligner [86] to align the text material to the stimulus waveforms, providing us with onset times of phonemes comprising the text. These were manually corrected using Praat [85] and subsequently transformed into a 23-dimensional binary articulatory feature space ("Art" [12]; see Figure S7). For this, we generated 23 time-series of zeros at a sampling rate of 40 Hz and inserted unit impulses at the onset times of phonemes corresponding to the respective articulatory feature. Finally, we discarded the information about phoneme identity to obtain a one-dimensional binary feature space of phoneme onsets ("PhOn").

Our set F_{MEG} of employed feature spaces then consisted of the following combinations: $F_{MEG} = \{\text{Env}, \text{Sg}, \text{Sg\&Deriv}, \text{Sg\&Gabor}, \text{Sg\&PhOn}, \text{Sg\&Art}, \text{Control}\}$, where Control was a combination of Sg, Deriv and Art. We downsampled the acoustic feature spaces to 40 Hz and z-scored all feature spaces prior to modeling.

Mapping from stimulus to MEG

To perform a linear mapping from our feature spaces to the recorded MEG signals, we used ridge regression [89] in a 6-fold nested cross-validation framework [44]. This allowed us to tune hyperparameters controlling the temporal extent and the amount of L2 regularization of the ridge models as well as the amount of regularization of the sensor covariance matrices and the coordinates of positions in source space for the beamformer spatial filters in the inner folds, yielding data-driven optimized models for each feature space, hemisphere and participant.

Linear model

The single-subject linear model we employed can be formulated in discrete time as:

$$\hat{r}_{\overline{c},\lambda_{source}}(t) = \sum_{\upsilon} \sum_{\tau = t_{Min}}^{t_{Max}} w(\upsilon, \tau) s(\upsilon, t - \tau)$$

Here, $\hat{r}$ denotes the neuronal response as obtained with a spatial filter with maximum gain at the vector $\overline{c}$ of coordinates (X, Y, Z) in source space and a regularization of the sensor covariance matrix of λ_{source}. Further, s is a representation of the stimulus in a given feature space, possibly multidimensional with dimensions υ. Finally, w describes the filter weights across these dimensions and time lags τ ranging from t_{Min} to t_{Max}, where negative values refer to samples in the future of t and positive values refer to samples in the past of t.

To obtain these filter weights, we used the following closed-form solution:

$$w = \left(S^T S + \lambda_{L2} I\right)^{-1} S^T r_{\overline{c},\lambda_{source}}$$

Here, S denotes the lagged time series of the stimulus representation, each column consisting of a particular combination of lags τ and feature dimensions υ, organized such that neighboring feature dimensions populate neighboring columns within groups of

columns corresponding to time lags. The identity matrix I is multiplied with λ_{L2}, a hyperparameter adjusting the amount of L2 regularization. Larger values of λ_{L2} force the resulting weights w closer to zero and thus reduce overfitting.

For the joint feature spaces consisting of multiple subspaces, the temporal extent and L2 regularization was optimized individually for each subspace to obtain the best possible prediction performance. This meant that the matrix S was constructed as the column-wise concatenation of multiple submatrices with different numbers not only of feature dimensions υ but also of lags τ. Additionally, this meant that λ_{L2} here was a vector instead of a scalar, with as many elements as feature spaces in the joint space. Corresponding to the concatenation of S, different sections of the diagonal of the identity matrix were multiplied with the dedicated regularization parameters of the corresponding subspace.

We used an additional regularization for the Gabor feature space. We had observed that feature dimensions belonging to the group of fastest temporal modulation frequencies ω had noisy and small filter weights at long absolute temporal lags. Based on this, we concluded that the temporal extents τ chosen for this feature space were essentially a compromise of long optimal τ for feature dimensions of slow ω ("Gb-Low") and short optimal τ for feature dimensions of fast ω ("Gb-Hi"). To remedy this problem, we assigned the usual τ to the group of slowest ω and added additional τ hyperparameters for the group of fastest ω. The τ of the central ω were then spaced proportionally to the mean auto-correlation times (ACT) of the corresponding groups of feature dimensions of this stimulus representation. We defined the ACT as the shortest lag where the normalized and absolute auto-correlation dropped below a value of 0.05. This allowed the optimization algorithm to pick long τ for feature dimensions of slow ω and short τ for feature dimensions of fast ω.

Nested cross-validation and hyperparameter tuning

To make data-driven optimal choices for the range of lags τ defined by t_{Min} and t_{Max}, the amount of L2 regularization λ_{L2}, the coordinates in source space as well as the amount of regularization of the sensor covariance matrices λ_{source}, we used nested cross-validation. Specifically, this means that we split our stimulus and response data in six portions of equal durations. Two loops then subdivided the data into training, tuning and testing sets. In each iteration, an outer loop assigned each of the six portions to be the testing set. Additionally, in each iteration of the outer loop, a full run of an inner loop was performed, assigning four portions to be the training set and the remaining portion to be the tuning set. This resulted in a total of 30 different assignments of portions to different sets. With this framework, we first picked a certain combination of hyperparameters and computed the corresponding weights w, the elementary parameters, using the training set. The resulting filters were convolved with the stimulus of the tuning set to obtain predictions $\hat{r}$ which we correlated with the observed responses r to obtain the tuning performance. This was repeated 200 times with different combinations of the hyperparameters.

These combinations were chosen by a recent black-box optimization algorithm, Bayesian Adaptive Direct Search [BADS] [45]. BADS uses Gaussian Processes to construct a computationally cheap internal model of the multidimensional performance landscape using already available evidence and smoothness assumptions. As the computationally relatively costly linear models are evaluated across iterations, more evidence about the true performance landscape builds up which is used to update the internal model, i.e., assumptions about the smoothness and shape of the performance landscape at hyper-parameter combinations not yet evaluated. The internal model is used to update an acquisition function, whose maximum determines which combination of hyperparameters would be most informative to evaluate next in order to find the global optimum of the performance landscape. While this algorithm is not guaranteed to find the optimal combination, i.e., it is possible that it gets stuck in local optima, it has been shown to outperform other black-box optimization algorithms on datasets typical for cognitive neuroscience [45]. The values at which the hyperparameters were initiated as well as the ranges to which they were constrained are shown in Table S1.

Once all iterations of an inner loop were finished, we averaged the hyperparameter choices of all inner folds. We then retrained the elementary model parameters with stimulus and response data corresponding to these averaged hyperparameters on all five possible assignments of data portions to training sets in the current outer fold. We subsequently averaged the elementary parameters across inner folds and used the resulting weights to perform a prediction on the test set of the current outer fold. This was repeated for all outer folds to obtain a number of test set predictions corresponding to the number of outer folds.

As the optimization procedure was not guaranteed to find the optimal combinations of parameters, a crucial quality control of our approach was to check the amount of variance across parameter choices. High degrees would e.g., reflect that the optimization algorithm would get stuck in local optima, or that the respective parameter was of minor importance for the model performance. Low degrees on the other hand would demonstrate that the black-box optimization would converge on the same choice.

For the positions in source space, we found the overall amount of variation to be rather small (Figure S2A). In the worst case (Figure S2B), the source locations were scattered within a range of 3.06 cm, the median of this range was 0.61 cm (Figure S2C), only slightly above the amount we allowed the participants to move in the scanner. In the best case, the range was only 0.23 cm.

We were also interested if our optimization would consistently pick distinct locations in source space for different feature spaces. To evaluate this, we computed the silhouette index. As a measure of the consistency of a clustering, it relates the similarity of data within a given class to the similarity of data outside of that given class and is bound between −1 and + 1. For the optimized source positions of each outer fold o of the set of outer folds O and each feature space f of the set of feature spaces F, we computed the silhouette index $s(o_f)$ using the following formula:

$$s(o_f) = \frac{b(o_f) - a(o_f)}{\max(a(o_f), b(o_f))}$$

Here, $a(o_f)$ denotes the average Euclidean distance between the source position chosen in the outer fold o and the source positions chosen in $O \setminus$ for that feature space f, while $b(o_f)$refers to the minimum of average distances between the source position chosen in the outer fold o for feature space f and source positions chosen for all outer folds in O for all feature spaces in $F \setminus f$.

Across feature spaces and hemispheres, we found results that were mostly inconsistent across participants (Figure S2D). Specifically, we observed participants for whom the assignment of chosen source positions to feature spaces was appropriate as reflected by silhouette indices close to 1, but also participants for whom this assignment was inappropriate as reflected by silhouette indices close to -1. In sum, on a group level and across feature spaces, there was no clear relationship between the choices of positions in source space and the feature space used to model the MEG responses. Overall, this suggests that while there was no direct and robust mapping of feature spaces to source positions, the optimization of the source positions tended to converge on relatively small regions within a participant.

The choices of optimal hyperparameters for the beamformer spatial filter did not differ substantially across feature spaces (Figure S2E). While we observed a relatively high degree of variance in optimal choices across participants, we found the choices to be relatively consistent within one hemisphere of a participant and across feature spaces as indicated by relatively high intra-class correlation coefficients across participants with outer folds and feature spaces as different measurements for the left (0.96) and right (0.88) hemispheres. However, we observed a pronounced difference between left and right ACs, with a higher level of regularization for the left AC. Here, in some cases the optimal values even bordered on the boundaries we chose for the hyperparameter, suggesting that in some cases, even higher values could have been optimal.

For the temporal extent, we found that this optimization resulted in characteristic temporal extents for each feature (sub-)space (Figure S3A). For example, for the combination of articulatory features and log-mel spectrograms the optimization algorithm consistently found shorter temporal extents for the articulatory features than for the log-mel spectrogram. Pooled across participants, we observed very similar patterns in left and right ACs.

For the L2 regularization, we again found that the optimization found characteristic values to be optimal for each feature (sub-) space. Specifically, for lower dimensional feature (sub-)spaces the amount of L2 regularization seemed to be less critical, yielding flat distributions. However, for higher-dimensional (sub-)spaces, a higher value of regularization seemed to be beneficial (Figure S3B). This was especially the case for the combination of articulatory features and the log-mel spectrogram, for which the distributions for the two subspaces clearly differ.

QUANTIFICATION AND STATISTICAL ANALYSIS

Model comparisons

Bayesian Hierarchical Modeling of performances

In an initial evaluation of the encoding models, we wanted to statistically compare the predictive performance from models using different feature spaces, obtained from multiple participants. Similar situations often arise in neuroimaging and are usually complicated by small raw effect sizes across conditions in the presence of much larger between subject variability. A promising way to address this is provided by hierarchical models, which allow to maintain sensitivity to effects of interest in these cases. To evaluate the model performances r in both hemispheres h for each outer fold b of all participants i and focus on the differences between the m different feature spaces f, we used a Bayesian hierarchical model with a zero intercept, participant-independent and participant-specific effects for each feature space as well as effects specific to each combination of participants and folds, participants and hemispheres as well as hemispheres and feature spaces. This allowed us to assess posterior distributions of the beta estimates of the means of each level of the categorical variable feature space. To implement this model, we used the brms package [54] within the R computing environment [81]. Specifically, the chosen package implements a user-friendly interface to set up Bayesian hierarchical models using stan [90]. We used Markov chain Monte-Carlo sampling with four chains of 4000 iterations each, 1000 of which were used for their warmup. The priors for standard deviation parameters were not changed from the default values, i.e., half-student-t distributions with 3 degrees of freedom, while we used weakly informative normal priors with a mean of 0 and a variance of 10 for the effects of individual feature spaces. The model can be described with the following formula:

$$
\begin{aligned}
r_n &\sim \mathcal{N}\left(\mu_n, \sigma^2\right) \\
\sigma &\sim |t(3, 0, 10)| \\
\mu_n &\sim \beta_{i:f[n]} + \beta_{i:b[n]} + \beta_{i:h[n]} + \beta_{h:f[n]} \\
&\quad + \beta_{f_1[n]} + \ldots + \beta_{f_m[n]} \\
\left(\beta_{i:f[n]}, \beta_{i:b[n]}, \beta_{i:h[n]}, \beta_{h:f[n]}\right) &\sim \mathcal{N}\left(0, \sigma^2_{\beta_{int}}\right) \\
\sigma_{\beta_{int}} &\sim |t(3, 0, 10)| \\
\beta_{f_1[n]} &\sim \mathcal{N}(0, 10) \\
&\vdots \\
\beta_{f_m[n]} &\sim \mathcal{N}(0, 10)
\end{aligned}
$$

To compare the resulting posterior distributions for several parameter combinations of interest, we evaluated the corresponding directed hypotheses using the *brms* package: $\beta_{f_a} - \beta_{f_b} > 0$, for all possible pairwise combinations of feature spaces, and obtained the ratio of samples of the posterior distributions of differences that were in line with the hypothesis.

Partial Information Decomposition

Besides directly comparing the raw predictive power of models across feature spaces, we were also interested in characterizing the detailed structure of predictive information carried in the different feature spaces. Since we were particularly interested in discovering to what degree the contributions of the annotated feature spaces can be explained with contributions of acoustic feature spaces, we thus asked to what degree their predictions contained the same information about the observed MEG (redundancy, or shared information) or to what degree their contributions were distinct (unique information). In information theory, this is possible within the framework of Partial Information Decomposition (PID) [55, 91]. This can be seen as a further development of the concept of interaction information [92] or co-information (defined equivalently but with opposite sign). Considering the case where we have two source variables (for example test set predictions from different models, $\widehat{r}_{M1}$ and $\widehat{r}_{M2}$) and a single target variable (for example the observed test set MEG time course, r), co-Information can be thought of as the set intersection of the two source-target MI values (i.e., the predictive information common to the two considered models). It is calculated as the difference between the sum of the individual source-target MIs and the full joint MI when considering both sources together:

$$\mathrm{CoI} = \mathrm{MI}(\widehat{r}_{M1}, r) + \mathrm{MI}(\widehat{r}_{M2}, r) - \mathrm{MI}([\widehat{r}_{M1}, \widehat{r}_{M2}], r)$$

If both sources provide the same information about the target then

$$\mathrm{CoI} = \mathrm{MI}(\widehat{r}_{M1}, r) = \mathrm{MI}(\widehat{r}_{M2}, r) = \mathrm{MI}([\widehat{r}_{M1}, \widehat{r}_{M2}], r)$$

which quantifies in this case fully redundant overlap in information content. However, it is possible that

$$\mathrm{MI}([\widehat{r}_{M1}, \widehat{r}_{M2}], r) > \mathrm{MI}(\widehat{r}_{M1}, r) + \mathrm{MI}(\widehat{r}_{M2}, r)$$

This results in a negative value for co-information and this sort of super-additive predictive effect is termed synergy.

Crucially however, co-information measures only the difference between redundancy and synergy, i.e., a net effect [55]. In the presence of equally strong synergistic and redundant contributions, co-information is zero. Therefore, co-information does not provide a way to quantify information provided uniquely by a single source.

The PID framework provides a solution to this problem. We used a recent implementation based on common change in surprisal (I_{ccs}) [46] which has previously been applied within a neuroimaging context [93]. The crucial step in a PID is to quantify redundancy, since once this is done, the other quantities (unique information and synergy) can then be inferred via a lattice structure [55]. For the redundancy measure I_{CCS}, pointwise co-information is considered.

MI can be quantified at the pointwise level (i.e., at specific values of the underlying variables): MI is defined as the expectation of pointwise MI (PMI) over all values of both variables and is non-negative. PMI on the other hand is a signed quantity. When it is positive it indicates those two particular values of the considered variables are more likely to occur together than would be expected if the variables were independent. When it is negative, it indicates that those two particular values are less likely to co-occur than in the independent case. Positive PMI can be interpreted as redundant entropy, while negative PMI is synergistic entropy [94]. Negative PMI values have also been termed misinformation [91], since they correspond to a case where a Bayes optimal gambler who was betting on the outcome of one variable based on observation of the other would actually do worse (on that particular observation) than if they ignored the observation.

In regression terms, negative PMI relates to values that, were they to occur in the data, would have large absolute residual from the regression line (i.e., deviate from the overall relationship), while positive PMI occurs for values that would be close to the regression line (i.e., following the overall relationship).

Similarly, pointwise co-information can be considered as quantifying the set theoretic intersection of PMI values from two sources. Two conditions have to be fulfilled in order for a pointwise co-information term to contribute to I_{CCS} redundancy: (I) both sources have PMI about the target with the same sign and (II) the pointwise co-information of these three variables is of the same sign as the two PMI values. This allows to quantify pointwise contributions of the sources about the target which can be unambiguously interpreted as redundant or overlapping contributions. A crucial advantage of this redundancy measure as opposed to other PID implementations is that it measures the overlap at the pointwise level and therefore can be interpreted as a within sample measure of redundant prediction, directly linked to the decoding interpretation of MI. This is essential for the comparison of predictive models as we consider here, for which redundancy measures which ascribe redundancy to sources even when they predict the target on disjoint sets of samples would be inappropriate [46].

This implementation of PID does not provide a non-negative decomposition. For example, negative unique information values are possible and they reflect a situation where there are pointwise misinformation terms that are unique to one source-target relationship [46; see Table 7]. In our application, negative unique information means there are time periods where one model mis-predicts, i.e., that combination of model prediction and MEG values is less likely to occur than if the model and prediction were shifted randomly, while the second model does not. In other words, there is a time window where that model is uniquely unhelpful for predicting the MEG signal, even though, of course, on average over time, it does have predictive value. In cases where there is negative unique information in the predictions of one model whose marginal MI about the MEG values is being used to normalize the redundancy values, it is therefore possible to obtain normalized redundancy ratios > 1.

We here performed PIDs for each combination of outer fold predictions of the annotated feature space with those of the acoustic feature spaces as sources and the recorded MEG as targets. Critically, we retrained all models with fixed hyperparameters of regularization of sensor covariance matrices and coordinates in source space to those previously chosen as optimal in the inner folds when training the model based on the Sg&Art feature space. This way, we gave the Sg&Art feature space the best chances to achieve maximal unique information. To compute the respective information theoretic quantities with these continuous variables, we transformed the variables to be standard normal while preserving rank relationships by calculating the empirical cumulative density function (CDF) value at each data point and applying the inverse standard normal CDF [43] prior to running I_{CCS} PIDs for Gaussian variables via Monte Carlo integration [46]. To interpret the raw values of the PIDs, we divided them by the marginal MI of the benchmark articulatory feature space prediction about the observed MEG. The normalized redundancy then represents the proportion of the predictive information of the benchmark model which is available also from the tested acoustic feature model.

To evaluate the results across folds, hemispheres, participants and feature spaces, we used Bayesian models similar to those used for the evaluation of the performances. The corresponding model can be described as follows:

$$\frac{red_n}{MI_n} \sim \mathcal{N}\left(\mu_n, \sigma^2\right)$$

$$\sigma \sim |t(3, 0, 10)|$$

$$\mu_n \sim \beta_{i:f[n]} + \beta_{i:b[n]} + \beta_{i:h[n]} + \beta_{b:f[n]}$$

$$+ \beta_{f_1[n]} + \ldots + \beta_{f_{m-1}[n]}$$

$$\left(\beta_{i:f[n]}, \beta_{i:b[n]}, \beta_{i:h[n]}, \beta_{h:f[n]}\right) \sim \mathcal{N}\left(0, \sigma^2_{\beta_{int}}\right)$$

$$\sigma_{\beta_{int}} \sim |t(3, 0, 10)|$$

$$\beta_{f_1[n]} \sim \mathcal{N}(0, 10)$$

$$\vdots$$

$$\beta_{f_{m-1}[n]} \sim \mathcal{N}(0, 10)$$

For the ratios of unique information, we concatenated the unique information of both competing sources x and y in all comparisons to a single response variable and changed the modeling approach to include predictors for unique information of both sources in all $m-1$ comparisons.

$$\frac{unq_n}{MI_n} \sim \mathcal{N}\left(\mu_n, \sigma^2\right)$$

$$\sigma \sim |t(3, 0, 10)|$$

$$\mu_n \sim \beta_{i:f[n]} + \beta_{i:b[n]} + \beta_{i:h[n]} + \beta_{h:f[n]}$$

$$+ \beta_{unqx_{f_1}[n]} + \ldots + \beta_{unqx_{f_{m-1}}[n]}$$

$$+ \beta_{unqy_{f_1}[n]} + \ldots + \beta_{unqy_{f_{m-1}}[n]}$$

$$\left(\beta_{i:f[n]}, \beta_{i:b[n]}, \beta_{i:h[n]}, \beta_{h:f[n]}\right) \sim \mathcal{N}\left(0, \sigma^2_{\beta_{int}}\right)$$

$$\sigma_{\beta_{int}} \sim |t(3, 0, 10)|$$

$$\beta_{unqx_{f_1}[n]} \sim \mathcal{N}(0, 10)$$

$$\vdots$$

$$\beta_{unqx_{f_{m-1}}[n]} \sim \mathcal{N}(0, 10)$$

$$\beta_{unqy_{f_1}[n]} \sim \mathcal{N}(0, 10)$$

$$\vdots$$

$$\beta_{unqy_{f_{m-1}}[n]} \sim \mathcal{N}(0, 10)$$

The resulting values of synergy were very low. We thus wanted to assess to which degree the observed synergy could only be obtained with intact predictions from the benchmark articulatory features, or to which degree it could also be observed when the benchmark's predictions were randomly permuted. We performed circular shifts of the predictions based on the Sg&Art features by a random number of samples, where the random number was constrained to be at least 200 samples and maximally the number of available samples minus 200 samples to avoid temporal autocorrelation. We computed PIDs of 1000 of these permutations. We then defined noise thresholds as the 95th percentile of the 1000 maximum values found in permutations across feature spaces, sources and outer fold test sets and calculated the fraction of data points (outer fold test sets, sources) within each participant and feature space. To also compare unique information of both sources and redundancy values to such noise thresholds, we repeated this process, shuffling predictions

based on the Sg&Art features for thresholds for the information unique to predictions based on the Sg&Art features and shuffling observed MEG time series for redundancy and information unique to predictions based on the competing feature spaces.

Phoneme-evoked dynamics

A recent study reported that epoching EEG recordings from a story-listening paradigm according to the onsets of phonemes allowed the decoding of four classes of phonemes, so-called manners of articulation, from the resulting event-related potentials [22]. We aimed to first replicate this finding with our MEG data and second assess to which degree our linear encoding models could account for this phenomenon.

We computed "Phoneme-Related Fields" (PRFs) using the 34562 phoneme presentations we had previously identified in our stimulus material. For this, we mapped the set of phonemes to manners of articulation as specified by Khalighinejad et al. [22; see Figure S7 for a mapping table]: Plosives, fricatives, nasals and vowels. We then epoched the continuous MEG data for a time range from $-0.1s - +0.6s$ around phoneme onsets, binned it across epochs for each time point using four equipopulated bins and computed mutual information between the MEG data and the four manners of articulation.

To ensure that we would capture the maximum effect of the MI, we delegated the choice of source positions for the left and right hemispheres as well as sensor covariance regularizations to the BADS algorithm similarly as before (Figure S4). However, this time we optimized the source model parameters with respect to the sum of MI of observed MEG data about the phoneme classes across time points. We then retrained our encoding models with the source model parameters fixed to these choices.

To assess the results of this optimization, we recalculated the maximum distance metric used in the assessment of the chosen source positions during our modeling, this time also including the positions found for optimal phoneme class decoding and plotted the difference to the previously obtained maximum differences (Figure S4A). The results reflected that still, all positions lay in STG, while for some participants, the positions found to be optimal for the PRF analysis were different from those obtained during the modeling.

Subsequently, we performed the same PRF analysis on the outer fold predictions of each feature space. We were then interested in the redundant and unique contributions of observed and predicted MEG to the MI about manners of articulation. We thus performed PIDs with observed and predicted PRFs as sources and the manners of articulation as the target, separately for each feature space, yielding phoneme-related redundancy as well as unique profiles.

Analysis of EEG dataset

To assess to which degree our main findings would generalize from our MEG to EEG data, we also performed an analysis of an openly available EEG story listening dataset [49]. This dataset is part of the data on which the effect of a gain of prediction performance of the combination of spectrograms and articulatory features over spectrograms alone was originally reported.

EEG preprocessing

We analyzed the 128 channel EEG recordings of a duration of 1 hour and 29 s of 13 participants. They had been acquired in 20 blocks of approximately equal duration at a sampling rate of 512 Hz using a BioSemi ActiveTwo system and downsampled to 128 Hz. We rereferenced the data to the average of two additional mastoid reference channels, spline interpolated noisy channels identified by visual inspection (mean number of noisy channels: 3.29 standard deviation: 4.19, pooled across participants), applied a fourth order forward-reverse butterworth high-pass filter with a cutoff frequency of 0.5 Hz and attenuated strong transient artifacts identified by visual inspection with a hamming window to have an absolute amplitude of 90% of the maximum of the absolute clean signal. Next, we z-scored individual blocks and winsorized the time series by replacing remaining artifacts with an amplitude stronger than ± 3 standard deviations by ± 3 and concatenated the individual blocks to single datasets. We then found unmixing matrices using the *runica* ICA algorithm. We identified artifactual components reflecting eye or heart activity and backprojected the unmixed data using mixing matrices where the artifactual components were removed. Finally, we downsampled the data to a sampling rate of 40 Hz.

Stimulus Transformations

In general, we reused the same pipeline to generate non-linear transformations of the stimulus as we had used for the stimulus of our MEG dataset. However, due to the high noise level of the EEG data, we decided to omit the high-dimensional Gabor feature space and focused on assessing if the acoustic feature space found to explain the performance gain of the benchmark articulatory features over spectrograms alone in the MEG dataset could do so in the EEG data as well. Additionally, we were interested in more faithfully reproducing the original results [10], where a spectrogram different from the log-mel spectrogram employed here had been applied. To do so, we generated a bank of 16 fourth order zero-phase butterworth bandpass filters with mel-spaced center frequencies (250, 402, 577, 780, 1015, 1288, 1605, 1971, 2396, 2888, 3459, 4121, 4888, 5777, 6807 and 8001 Hz), where the cutoff frequencies were defined as half of the distances to the neighboring center frequencies. The absolute values of the Hilbert transform of the output of these filters served as an approximation to the spectrogram used in the original publication ("Sg16"). Moreover, we were interested to which degree possible differences between the performances achieved with this spectrogram compared to our log-mel spectrogram were attributable to a compressive nonlinearity [19] included in the latter. We therefore generated an additional spectrogram ("Sg16c") where we raised the values of Sg16 to the power of 0.3. This gave us a set of feature spaces $F_{EEG} = \{Env, Sg16, Sg16c, Sg, Sg16\&Deriv16, Sg16c\&Deriv16c, Sg\&Deriv, Sg16\&PhOn, Sg16c\&PhOn, Sg\&PhOn, Sg16\&Art, Sg16c\&Art, Sg\&Art, Control\}$

Forward Modeling

To keep the results comparable to the original publication, we performed ridge regressions to model responses at the 12 electrodes whose performances were reported in the main result of the original publication (B28, B29, B30, C3, C4, C5, D3, D4, D5, D10, D11, D12) using the function "mTRFcrossval.m" from the mTRF toolbox [89]. However, we implemented a small change that allowed us to

do a nested crossvalidation to tune the regularisation hyperparameter λ_{L2}. We trained models on 18 of the 20 available blocks, picked the λ_{L2} that resulted in the best prediction performance on a validation block and evaluated the test performance on the remaining block. This procedure was rotated such that each block served as the test set once. We specified the range of λ_{L2} values as $\{0.1\,k\,|\,k\,\epsilon[-25...60]\}$, over which the function performed an exhaustive grid search where the extreme values were never chosen. For the parameters of temporal extent, we used the same values as in the original publication, i.e., $t_{Min} = -0.1$ and $t_{Max} = 0.4$ seconds.

Model Comparisons

The model comparisons employed here were largely the same as for the MEG data.

To evaluate the test set prediction performances of the forward models, we used the same Bayesian modeling approach as we had used for the analysis of the MEG data.

We also performed the same PID analysis with model predictions as sources and observed EEG time-series as targets and evaluated its performances with the same Bayesian models as we had used for the MEG analysis. However, due to the higher noise level, we only considered data points where the MI of predictions based on the benchmark articulatory feature space and observed time-series surpassed a noise threshold defined as the 95th percentile of MI values obtained from time shifted permutations, corrected across electrodes using maximum statistics. Additionally, to account for the skewed distributions of the ratios of PID quantities normalized by the marginal MI of the predictions based on the benchmark articulatory feature space and the observed MEG, we used a log-normal response family for the Bayesian modeling and considered the posterior distributions of the medians of the effects of interest. We repeated the computation of noise thresholds as described for the MEG data. Finally, we performed the same analysis of phoneme evoked responses on the set of electrodes used for the modeling as we had performed on the MEG data.

DATA AND SOFTWARE AVAILABILITY

Anonymized MEG and structural MRI data as well as custom code are available on demand by emailing the Lead Contact.

Current Biology, Volume *29*

Supplemental Information

Simple Acoustic Features Can Explain Phoneme-Based Predictions of Cortical Responses to Speech

Christoph Daube, Robin A.A. Ince, and Joachim Gross

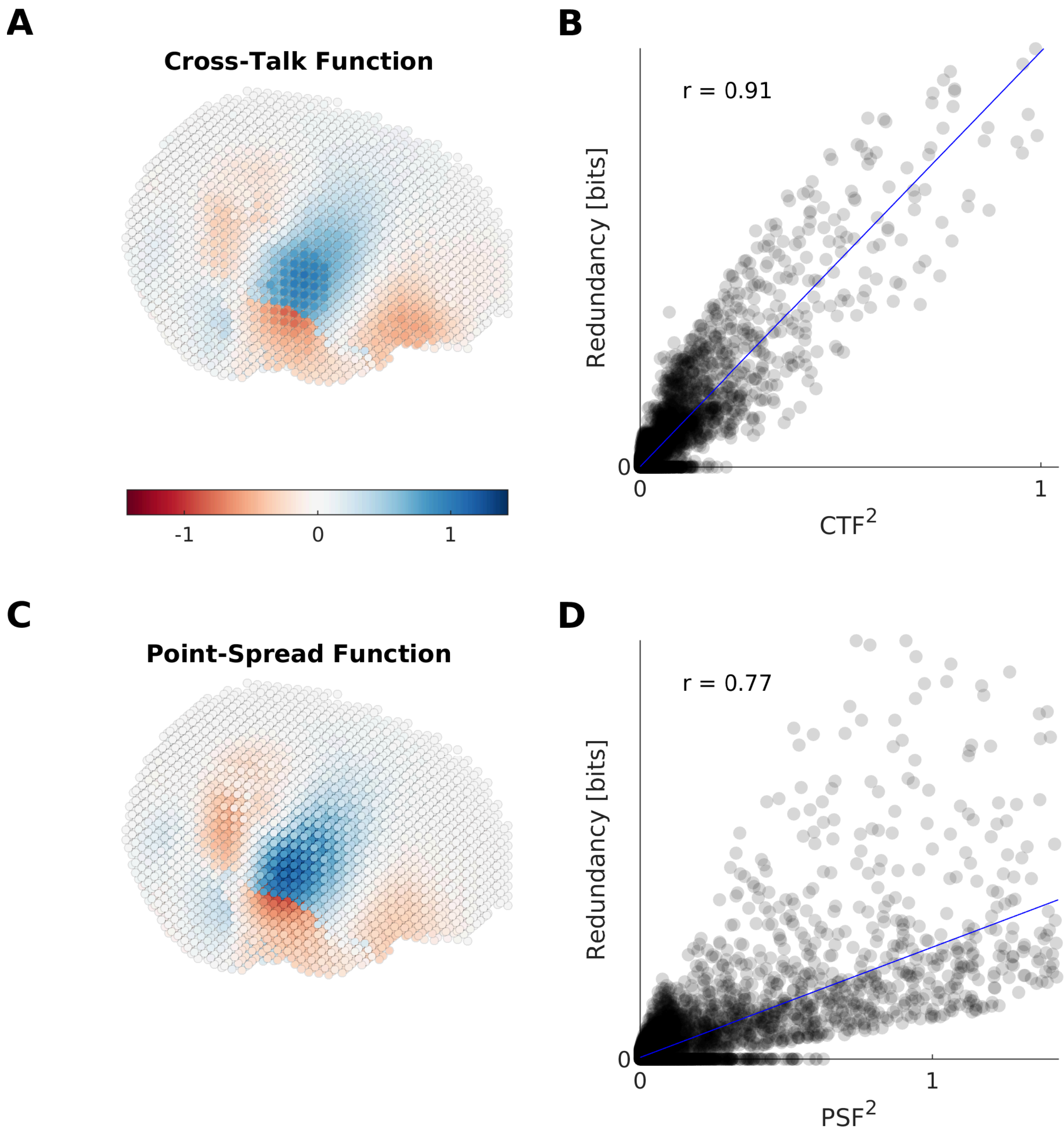

Figure S1: Redundancy is related to Cross-Talk and Point-Spread Functions (related to Figure 2). **(A)** Cross-Talk Function in one exemplary participant (same as in figure 2B). It shows how activity at different grid points leaks into estimates of activity queried at grid point of interest, here at a right AC dipole. **(B)** Relationship of squared Cross-Talk Function (CTF) and Redundancy in exemplary participant. Correlation in top left reports Pearson correlation. The mean of this correlation (Fisher-Z transformed before averaging and retransformed after averaging) was 0.75 (right AC, range: $[0.26, 0.91]$) and 0.68 (left AC, range: $[-0.09, 0.94]$). **(C)** Point-Spread Function in one exemplary participant. It shows how activity at grid point of interest, here at a right AC dipole, leaks into estimates of activity queried at other positions in source space. **(D)** Relationship of squared Point-Spread Function (CTF) and Redundancy in exemplary participant. Correlation in top left reports Pearson correlation. The mean of this correlation (Fisher-Z transformed before averaging and retransformed after averaging) was 0.60 (right AC, range: $[0.16, 0.83]$) and 0.51 (left AC, range: $[-.10, .87]$).

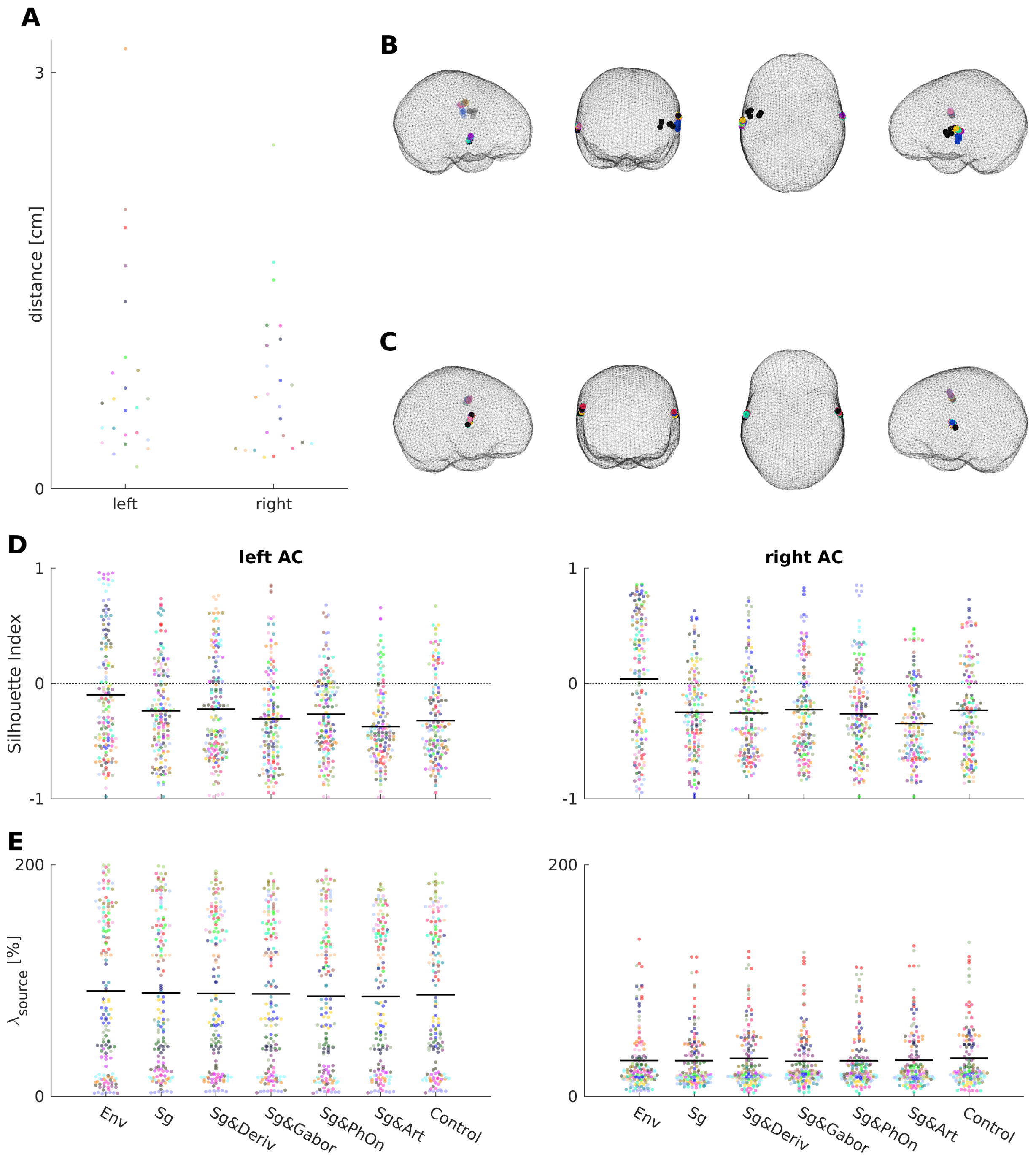

Figure S2: Hyperparameter choices for source model optimisation (related to Figure 3).
(A) Maximum distance between source positions used for test set prediction across all test sets and feature spaces. Each dot is one participant in the respective hemisphere, colour codes participants. **(B)** and **(C)** Positions of all test sets and feature spaces in meshes of individual brain volumes of two exemplary participants. **(B)** shows the participant with the largest maximum distance between chosen source locations and **(C)** the participant closest to the median of maximum distances between chosen source locations. Each dot is one test set, colour codes feature spaces. **(D)** Evaluation of spatial clustering of choices of source positions for different feature spaces using the Silhouette Index. Values close to 1 reflect that the optimisation procedure finds positions in source space that are highly simliar within feature spaces but dissimilar across feature spaces, lower values reflect that the found positions are randomly arranged in source space. Each dot is one test set of one participant, colour codes participants. Black lines denote pooled means. **(E)** Choices of beamformer regularisation hyperparameter λ_{source} for each feature space. Each dot represents one test set of one participant, colour codes participants. Black lines denote pooled means.

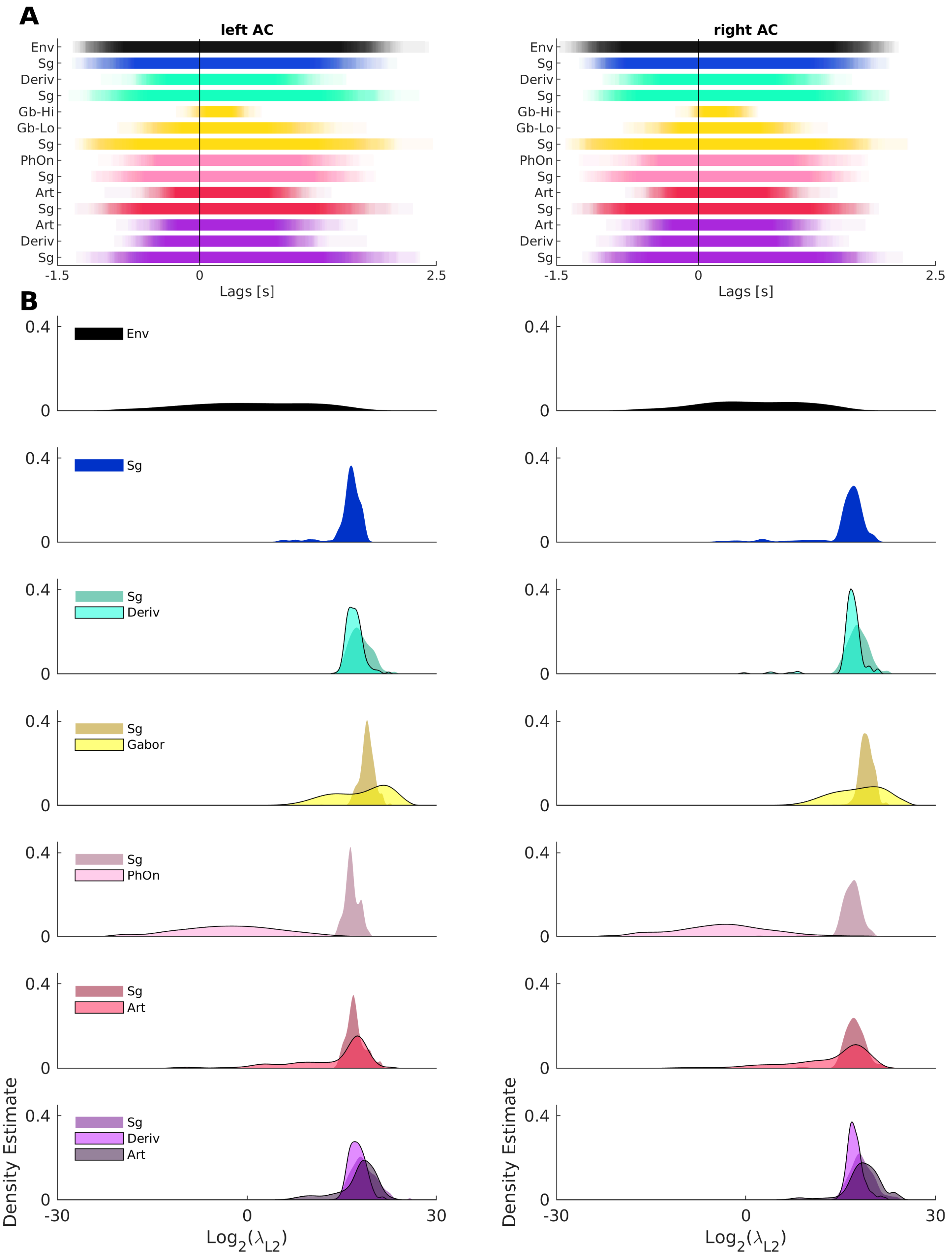

Figure S3: Hyperparameter choices of forward model optimisation differ systematically across feature (sub-)spaces (related to Figure 3).
(A) Choices of temporal extent hyperparameters for each feature (sub-)space. Shown are averages across inner folds used for test set predictions, pooled across participants. Values that were used in all cross-validated models of all participants are plotted as transparent bars ranging from $t_{Min} - t_{Max}$ such that the opacity codes for the number of participants and folds for which the temporal extent was chosen correspondingly. **(B)** Choices of L2 regularisation hyperparameters for each feature (sub-)space. Shown are distributions of choices averaged across inner folds used for test set predictions, pooled across participants. Colours code feature spaces.

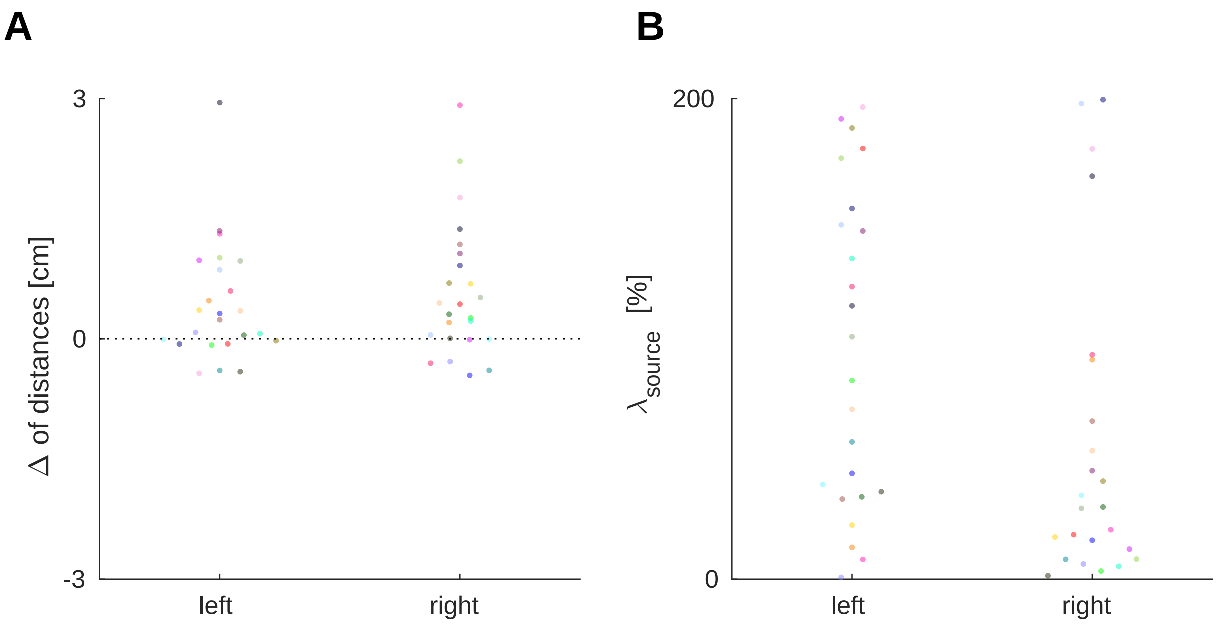

Figure S4: Hyperparameter choices for phoneme related field (PRF) analysis (related to Figure 5).
(A) Maximal euclidean distances of source positions when optimised with regard to model performances across all test sets and feature spaces subtracted from maximal euclidean distances of source positions when positions found when optimising with regard to PRF MI are included. **(B)** Results of optimising sensor covariance regularisation parameter with regard to PRF MI. Colour in **(A)** and **(B)** codes participants.

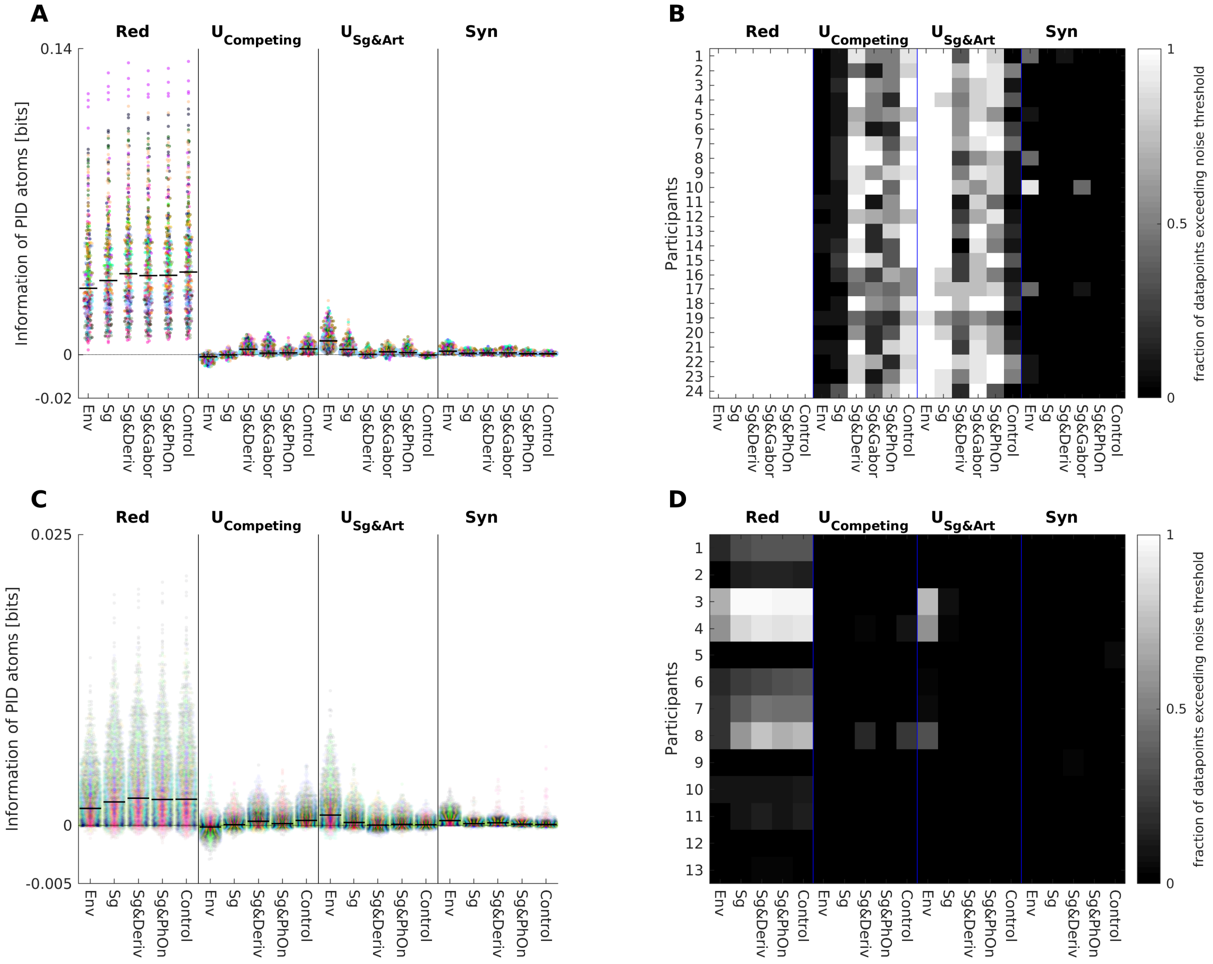

Figure S5: Raw values and comparison to noise thresholds of PID in EEG and MEG (related to Figures 4 and 6).

(A) Raw (unnormalised) PID values (Red: redundancy, U: unique information [of competing feature spaces and of benchmark articulatory feature space] and Syn: synergy) in MEG data from left and right AC. Each colour codes for a single participant, each dot is one test set. Pooled medians are indicated with black lines. **(B)** Comparison of PID values in MEG data to noise thresholds. Image plot shows the fraction of data points (sources, test sets) that exceeded the corresponding noise threshold in each participant and for each feature space and each PID atom. **(C)** Raw (unnormalised) PID values (redundancy, unique information of competing feature spaces, unique information of benchmark articulatory feature space and synergy) in EEG data from all 12 electrodes. Each colour codes for a single participant, each dot is one test set. Pooled medians are indicated with black lines. **(D)** Comparison of PID values in EEG data to noise thresholds. Image plot shows the fraction of data points (sources, test sets) that exceeded the corresponding noise threshold in each participant and for each feature space and each PID atom.

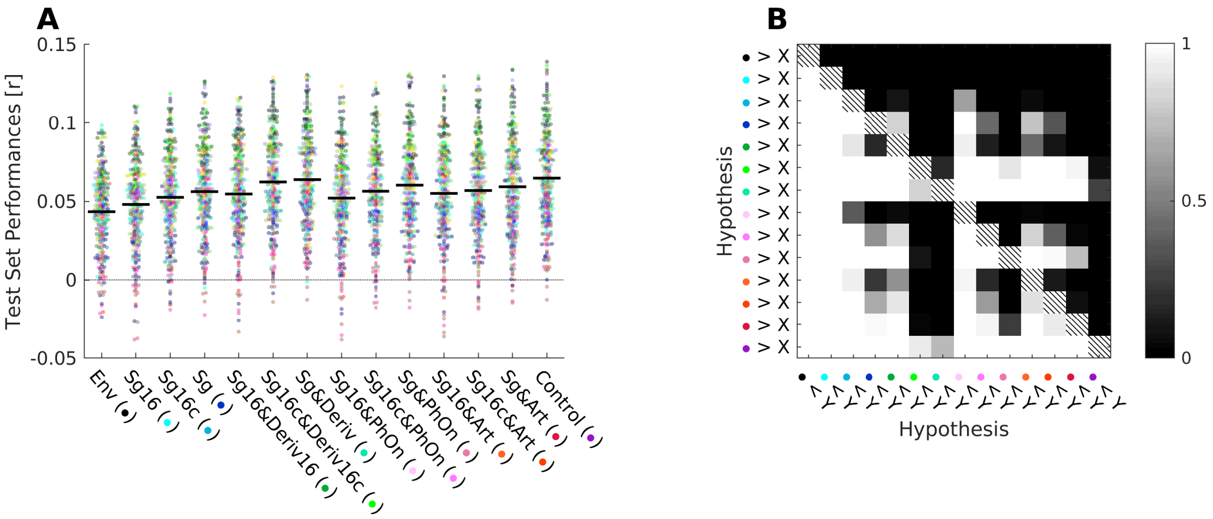

Figure S6: Comparison of 16 channel spectrogram, 16 channel spectrogram with compressive nonlinearity and log-mel spectrogram in EEG data (related to Figure 6).
(A) Raw test set performances of feature spaces. Each dot is one test set of one participant, averaged across electrodes. Colour codes participants. Pooled medians overlaid. Colours in x-axis labelling refer to feature spaces. **(B)** Percent of samples in favour of hypotheses of differences of beta estimates between all feature spaces. Hypotheses are colour coded using the same colour mapping as in x-axis labelling of **(A)**.

The performances obtained using Sg were in general higher than those obtained using $Sg16$, a 16-channel spectrogram modelled after the original study [1]. The combination of $Sg16\&Art$ failed to outperform Sg on its own ($f_{h_1} = 0.2318$).

The overall pattern of performances was largely similar regardless of using Sg or $Sg16$ as the spectrogram. In contrast to the results produced using Sg however, we found that the $Sg16\&Art$ combination outperformed the $Sg16\&PhOn$ combination ($f_{h_1} = 0.9675$).

To assess whether these differences were driven by the compressive linearity included in Sg, we additionally tested a version of $Sg16$ in which we raised its values to the power of 0.3 ("$Sg16c$"). Such nonlinearities are classically included in models of auditory processing, as early as the cochlea [2; 3; 4]. This tweak indeed resulted in a pattern of performances that was largely similar to that obtained with Sg: The combination of $Sg\&Deriv$ did not clearly outperform the combination of $Sg16c\&Deriv16c$ ($f_{h_1} = 0.8191$), and combining $Sg16c\&Art$ was not better than combining $Sg16c\&PhOn$ ($f_{h_1} = 0.6097$).

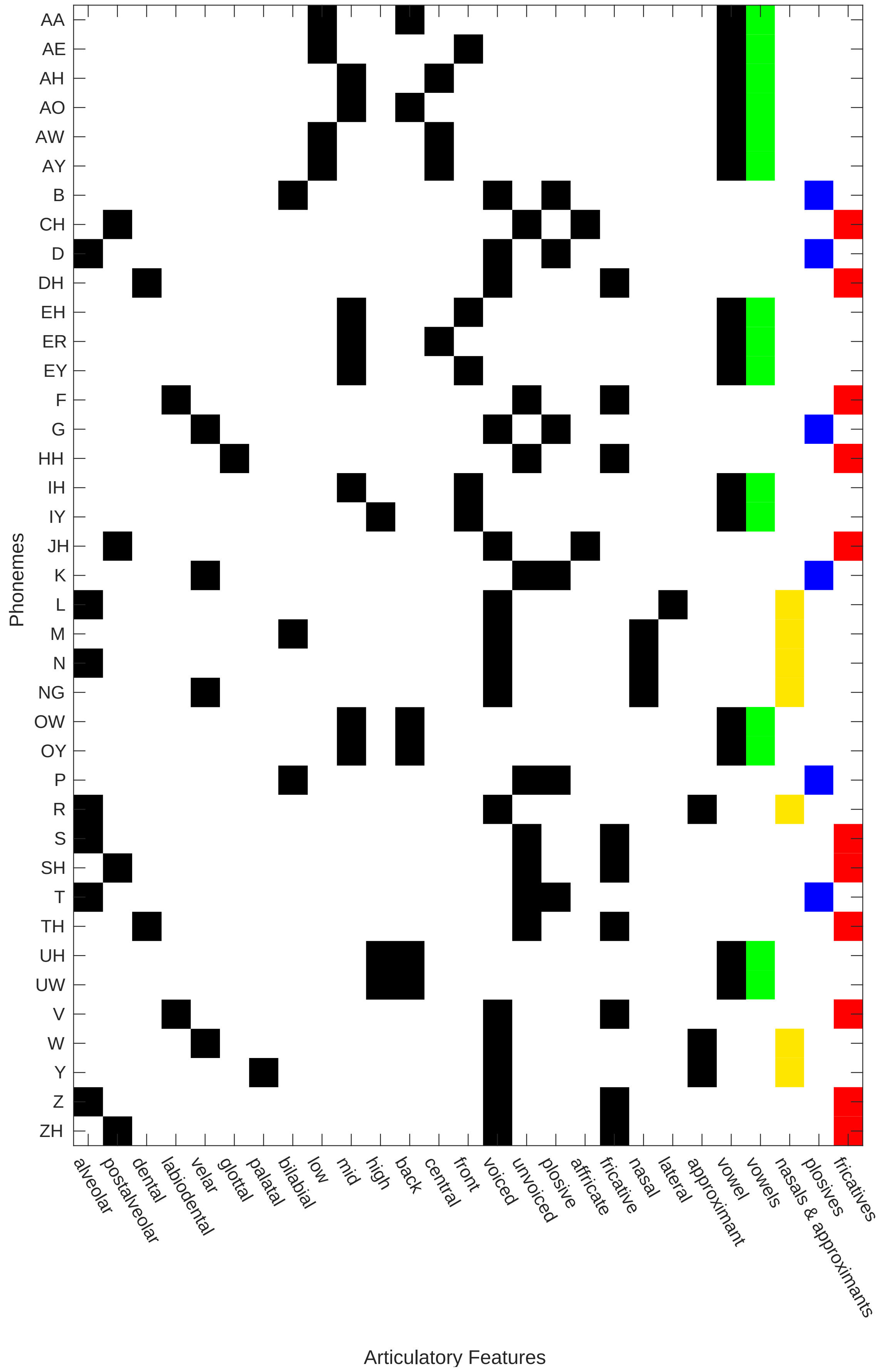

Figure S7: Mapping of phonemes to articulatory features and manners of articulation (related to STAR Methods).
Black and white part shows articulatory features used for forward modelling [1], coloured part shows manners of articulation used for decoding [5].

	initial value	lower boundary	upper boundary
$t_{Min}[s]$	-0.2	-1.5	.5
$t_{Max}[s]$	0.8	0.2	2.5
$\log_{2(\lambda_{L2})}$	19	-30	30
$\lambda_s[\%]$	30	0	200
X_{source}	$X_{max(R^2)\|hemisphere}$	$min(X_{volume\|hemisphere})$	$max(X_{volume\|hemisphere})$
Y_{source}	$Y_{max(R^2)\|hemisphere}$	$min(Y_{volume\|hemisphere})$	$max(Y_{volume\|hemisphere})$
Z_{source}	$Z_{max(R^2)\|hemisphere}$	$min(Z_{volume\|hemisphere})$	$max(Z_{volume\|hemisphere})$

Table S1: Initial values and boundaries for hyperparameters in BADS optimisation (related to STAR Methods).

Supplemental References

S1 Di Liberto, G.M., O'Sullivan, J.A., and Lalor, E.C. (2015). Low-Frequency Cortical Entrainment to Speech Reflects Phoneme-Level Processing. Current Biology *25*, 2457–2465.

S2 Chi, T. and Powen, R. (2005). Multiresolution spectrotemporal analysis of complex sounds. The Journal of the Acoustical Society of America *118*, 887–906.

S3 Verhulst, S., Altoè, A., and Vasilikov, V. (2018). Computational modeling of the human auditory periphery: Auditory nerve responses, evoked potentials and hearing loss. Hearing Research *360*, 55–75.

S4 Biesmans, W., Das, N., Francart, T., and Bertrand, A. (2017). Auditory-Inspred Speech Envelope Extraction Methods for Improved EEG-Based Auditory Attention Detection in a Cocktail Party Scenario. IEEE Transactions on Neural Systems and Rehabilitation Engineering *25*, 402–412.

S5 Khalighinejad, B., da Silva, G.C., and Mesgarani, N. (2017). Dynamic Encoding of Acoustic Features in Neural Responses to Continuous Speech. The Journal of Neuroscience *37*, 2176–2185.
